# The End of a 60-year Riddle: Identification and Genomic Characterization of an Iridovirus, the Causative Agent of White Fat Cell Disease in Zooplankton

**DOI:** 10.1534/g3.117.300429

**Published:** 2018-02-27

**Authors:** Elena R. Toenshoff, Peter D. Fields, Yann X. Bourgeois, Dieter Ebert

**Affiliations:** 1 Basel University, Department of Environmental Sciences, Zoology, Vesalgasse 1, CH-4051 Basel, Switzerland

**Keywords:** Cladocera, Crustacea, virus, iridovirus, genome, horizontal gene transfer, White Fat Cell Disease

## Abstract

The planktonic freshwater crustacean of the genus *Daphnia* are a model system for biomedical research and, in particular, invertebrate-parasite interactions. Up until now, no virus has been characterized for this system. Here we report the discovery of an iridovirus as the causative agent of White Fat Cell Disease (WFCD) in *Daphnia*. WFCD is a highly virulent disease of *Daphnia* that can easily be cultured under laboratory conditions. Although it has been studied from sites across Eurasia for more than 60 years, its causative agent had not been described, nor had an iridovirus been connected to WFCD before now. Here we find that an iridovirus—the Daphnia iridescent virus 1 (DIV-1)—is the causative agent of WFCD. DIV-1 has a genome sequence of about 288 kbp, with 39% G+C content and encodes 367 predicted open reading frames. DIV-1 clusters together with other invertebrate iridoviruses but has by far the largest genome among all sequenced iridoviruses. Comparative genomics reveal that DIV-1 has apparently recently lost a substantial number of unique genes but has also gained genes by horizontal gene transfer from its crustacean host. DIV-1 represents the first invertebrate iridovirus that encodes proteins to purportedly cap RNA, and it contains unique genes for a DnaJ-like protein, a membrane glycoprotein and protein of the immunoglobulin superfamily, which may mediate host–pathogen interactions and pathogenicity. Our findings end a 60-year search for the causative agent of WFCD and add to our knowledge of iridovirus genomics and invertebrate–virus interactions.

Infectious diseases affect almost all life forms on Earth and have been implicated as drivers of biodiversity and as the force behind the coevolution of hosts and infectious disease agents — a dynamic that has been theorized to explain many life phenomena, including high genetic diversity at resistance loci, the maintenance and evolution of sexual recombination, and sexual selection (Schmid-Hempel 2011). Despite years of study, however, we still have a rather rudimentary understanding of the diversity of parasites, including pathogens, that occur in natural host populations. In water fleas of the genus *Daphnia*, a model system for studying the ecology, evolution, epidemiology of infectious disease and host-parasite interactions, many *Daphnia* parasites and pathogens have been reported (Green 1974; Mangin *et al.* 1995; Decaestecker *et al.* 2003; Ebert 2005). However, few studies have reported viruses in the water fleas. This is surprising because the *Daphnia’s* habitat — standing freshwater — is conducive to viruses, protecting them from desiccation and UV light, facilitating transmission, and allowing for the buildup of local epidemics and transmission stage banks.

Two diseases with very similar symptoms have been described for the water flea *Daphnia magna* (Branchiopoda: Cladocera): one is an unnamed iridovirus (Bergoin *et al.* 1984; Vávra *et al.* 2016); the other is White Fat Cell Disease (WFCD) (Green 1957). In both diseases, the normally semitransparent host presents with extremely enlarged fat cells that are white and nontransparent, with a characteristic greenish, iridescent shine in reflected light. Infected animals carry almost no eggs in their brood-pouch (Figure 1). WFCD was first observed in *D. magna* in rock pool populations in southwestern Finland. Green (1957) postulated that the highly virulent infection was caused by a small coccoid bacterium and named it “White fat cell bacterium” and also “White bacterial disease” (WBD). The term “White bacterial disease” has since been picked up and used in many publications (for example (Green 1974; Ebert *et al.* 2000; Little and Ebert 2000; Van De Bund and Van Donk 2002; Decaestecker *et al.* 2003; Decaestecker *et al.* 2005; Ebert 2005; Coopman *et al.* 2014; Lange *et al.* 2014; Duffy *et al.* 2015; Panadian 2016) and the disease has been reported in England, Belgium, Finland, France, Netherlands (Green 1974; Little and Ebert 2000; Van De Bund and Van Donk 2002; Decaestecker *et al.* 2003; Decaestecker *et al.* 2005) and Israel (F. Ben-Ami, personal communication). Studies of WFCD in both the laboratory and the field have revealed that it is horizontally transmitted via waterborne transmission stages from dead hosts and is highly virulent (Ebert *et al.* 2000; Decaestecker *et al.* 2005; Coopman *et al.* 2014; Lange *et al.* 2014). That the disease agent was bacterial in nature was never questioned, despite the fact that numerous attempts to isolate the putative disease-causing bacterium and to obtain its sequence data failed (Dieter Ebert (unpublished)).

**Figure 1:**
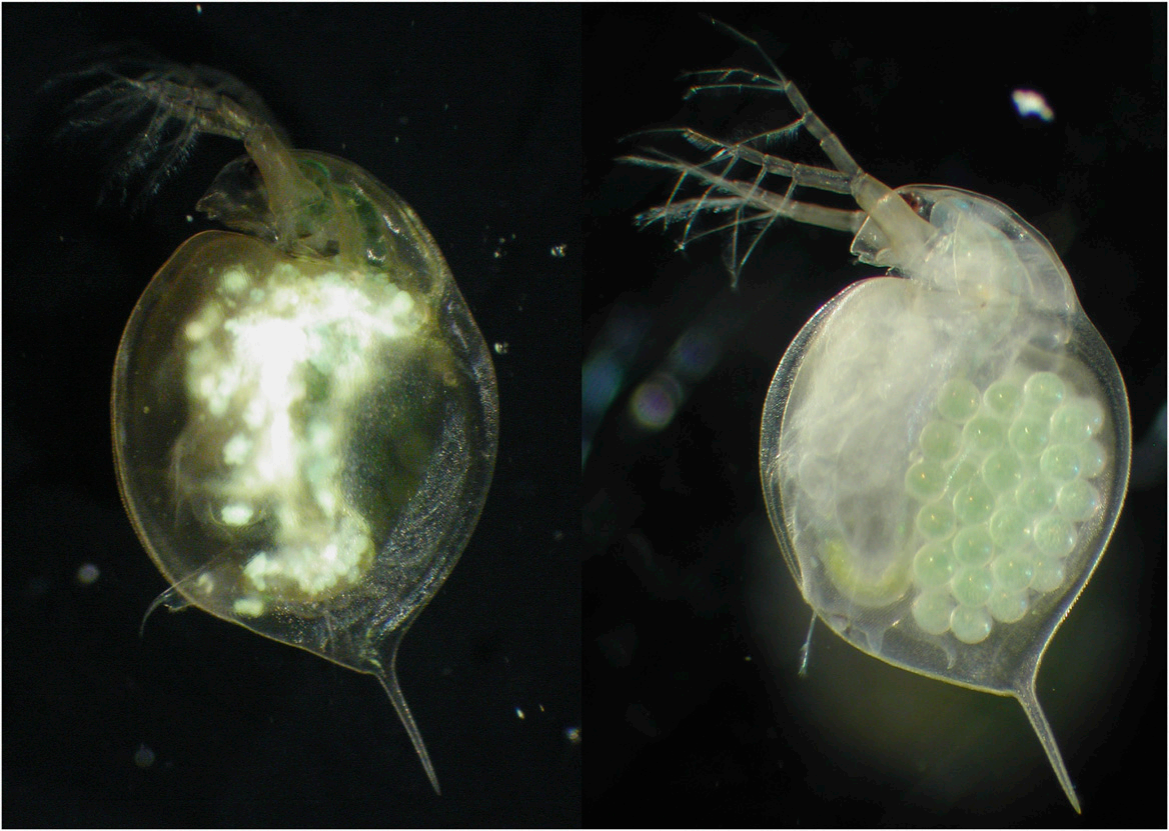
*D. magna* infected by Daphnia iridescent virus 1 (DIV-1) (left) and uninfected (right). The infected *D. magna* shows the typical symptoms of WFCD: white, shiny fat cells in the central part of the body and reduced fecundity.

Independent of the studies on WFCD, Bergoin *et al.* (1984) described *D. magna* individuals from Mediterranean salt-marshes in southern France with an infection phenotype similar to WFCD. Using electron microscopy (EM), he showed that these *D. magna* were infected with a virus most likely belonging to the *Iridoviridae*. In 2016, Vávra *et al.* (2016) published ultrastructural pictures of an iridovirus that caused the same symptoms in *Daphnia curvirostris* in the Czech Republic. Both studies compared their findings to a previously observed iridovirus in another Cladoceran species, *Simocephalus expinosus* (Federici and Hazard 1975) but did not link their results to the research on WFCD. The phenotypic similarity between WFCD and the iridovirus infections prompted us to investigate if an iridovirus might be the causative agent of WFCD. We here report that WFCD is indeed caused by an iridovirus that induces a clear pathology in its host, and that it is likely the same virus described earlier by Bergoin *et al.* (1984) as an iridovirus.

Iridoviruses are double-stranded DNA (dsDNA) viruses with comparatively large genomes. They are globally distributed and cause mild to lethal infections (Jancovich *et al.* 2012). Besides water fleas (*Cladocera)* (Federici and Hazard 1975; Bergoin *et al.* 1984; Vávra *et al.* 2016) and other crustaceans, they are reported in insects, mites, mollusks, annelids, and nematodes (Williams 2008) as well as in poikilothermic vertebrates (Williams *et al.* 2005; Jancovich *et al* 2012). As iridoviruses are economically and ecologically significant, especially in aquaculture, they are increasingly receiving research attention (Chinchar 2002; Zhang and Gui 2015; Epstein and Storfer 2016; Rijks *et al.* 2016). To date, 53 complete iridoviral genome sequences have been reported (NCBI, https://www.ncbi.nlm.nih.gov/; query date: July 4, 2016), although only one of these is associated with crustaceans, the Armadillidium vulgare iridescent virus (Piégu *et al.* 2014).

The aim of this study was to determine the causative agent of WFCD in *D. magna* and clarify its taxonomic position. We conducted ultrastructural analyses, sequenced the genome of this causative agent, and investigated the evolution of its genome and its genomic adaptation to *D. magna* using comparative genomics.

## Material and methods

### Ethics statement

The work in this study involved no human or vertebrate subjects, only small planktonic invertebrates belonging to the lower Crustacean (*Daphnia magna*). Collection and work with *Daphnia* in this study did not require permission. Our study did not involve the use or collection of endangered or protected species. License from our institution is not required to work with *Daphnia*.

### Material

The virus isolates used for the transmission experiment and the genome sequence in this study were collected from a natural rock pool population on the skerry island Spicarna (FI-SP1; 59°48’43.0”N 23°12’30.9”E), about 3 km from the site where Green (1957) first reported the disease. For the preparation of the infection assay we collected 20 infected animals and brought them to the lab where they were processed within two hours. Homogenates of infected D. magna were used to transmit the virus to an uninfected laboratory culture of a *Daphnia magna* clone previously isolated and cloned from the same sampling site. This infected culture served as the stock for the infection experiment. For the genome sequencing project, we collected about 200 infected animals and brought them to the lab where they were processed within two hours (using the procedure described below). The virus isolate used for the fluorescence and transmission electron microscopy originated from a different population (Great Britain, population GB-EK2: 55°41’51.58”N, 2°20’36.36”W). A population sample was cultured in our laboratory and infected females were used for microscopy. This virus is phenotypically and pathologically indistinguishable from the Finnish virus isolate.

Two *Daphnia magna* genotypes (=clones) used in the transmission experiment originated from the same sites as the virus (clones: Finland_1 and Finland_2). Nine additional *D. magna* clones from sites across Europe and Israel, covering the known geographic distribution of the virus, were also used. These clones originated from field isolates from Finland, Sweden, Hungary, Belgium, Great Britain, Switzerland, France, Greece and Israel. *D. magna* clones were kept in mass culture under controlled environmental conditions in 400-mL jars, at 20°, 16:8 h light:dark cycle, with green algae *Scenedesmus* sp. as the only food. Each clone had been started from a single offspring. The clonal replicates are genetically identical.

### Infection assay

Female juvenile *D. magna* from the 11 clones were placed individually in 100-mL jars with 20 mL artificial *Daphnia* medium (ADaM) modified from Klüttgen *et al.* (1994). We aimed for 30 animals per clone at the start of the experiment (11 host genotypes × 30 = 330 animals); however, due to problems raising some of the genotypes, we started the experiment with 283 females, with three genotypes contributing only 9, 15 and 21 animals. If enough females from a host genotype were available, we inoculated 20 with the virus suspension, while 10 were sham infected, otherwise proportionally less females.

To produce a virus suspension, 25 strongly infected *D. magna* were taken from our infected laboratory *D. magna* culture (see above). The animals were ground, diluted in artificial *Daphnia* Medium (A*Da*M) and strained through a filter with 0.2 µm pore size (Millipore) to ensure that the filtrates were free of bacteria, which are larger than 0.2 µm. The virus suspension was distributed across the treatment group (in total 202 animals). Controls (in total 81 animals) were sham infected with filtered ADaM only. Every second day, the animals were fed with 5 million cells green algae. After three days, the jars were topped-off with an additional 60 mL of ADaM. Over a period of 21 days, the animals were checked daily for infection and survival. In case of doubt, we used a dissecting microscope with reflected light to check for infection, as that made infections in the fat cells clearly visible. During the experiment, offspring were removed from the jars. A logistic regression (glm: infection ∼ hostclone, family = binomial(link=’logit’)) was calculated using the R software (R Development Core Team 2014).

### Fluorescence and transmission electron microscopy

To conduct the fluorescence microscopy, we took daphnids showing signs of WFCD (population GB-EK2) and cut them on a glass slide. We then mounted them with VECTASHIELD containing DAPI (4’,6-diamidino-2-phenylindole) (Vector Laboratories), and further examined them using a Leica CTR 5000 fluorescence microscope. For transmission electron microscopy, we cut daphnids showing signs of WFCD (population GB-EK2) and prefixed them in Karnovsky fixative for 1 h at room temperature. Pieces of the cut *Daphnia* were mounted with agar and fixed with Karnovsky fixative for 24 h. The samples were then fixed in 1% osmium tetroxide for 1 h 20 min and dehydrated in an increasing ethanol series (50- 100%). An *en bloc* staining with 2% uranyl acetate was performed at 70% ethanol. Specimens were treated with acetone and embedded in Epon 812 resin. Ultrathin sections were examined using the Philips CM100 electron microscope. Measurement of particles was done by ImageJ (Schneider *et al.* 2012).

### DIV-1 enrichment, DNA purification, and sequencing

*D. magna* collected in the field from the Finnish rock pool population (see above) were sorted to enrich the sample with animals that showed clear signs of WFCD infection (Figure 1). We followed two different approaches for obtaining genomic DNA. In one batch of about 50 animals, we enriched the sample with infected tissue by cutting off body parts that showed no visible signs of infections, *i.e.*, the head, the antenna, the carapace. The retained tissue was placed in a 2-mL screw-cap tube and submerged in RNA*later* solution (Sigma-Aldrich). The second batch of about 50 animals underwent an antibiotic treatment to reduce non-focal DNA sources, *i.e.*, bacteria of the microbiome and food particles. Individuals were treated for 72 h with three antibiotics (streptomycin, tetracycline, ampicillin) at a concentration of 50 mg/L each, with the antibiotic solution refreshed every 24 h. During this time, animals were fed dextran beads (Sephadex, size class ‘Small’, about 50 µm diameter, Sigma-Aldrich) at a concentration of 0.5 g/100 mL to aid in the expelling of gut contents. The surviving animals were moved out of antibiotic solution and into a 2-mL screw-cap tube. Excess fluids were removed using a sterile pipette, and RNA*later* solution was added. After 12 hr at 4°, the tubes were moved to a -20° freezer and stored at -80° until further processing.

Before extracting the DNA, we pipetted the RNA*later* solution away from the tissue. The tissue was then rinsed twice with DNA- and RNA-free, sterile water. Extraction buffer (Gentra Puregene Tissue Kit, Qiagen) was added to the tubes, and the tissue was ground using a sterile and DNA-free plastic pestle. The resulting solution was incubated overnight with Proteinase K at 55°. The RNA was degraded using RNase treatment for one hour at 37°. Protein removal and DNA precipitation, which was facilitated by the addition of glycogen (Sigma-Aldrich CAS# 9005-79-2), were conducted following the Gentra Puregene Tissue Kit (Qiagen) instructions. The resulting purified DNA was suspended in 40 µL of DNA hydration solution (Qiagen) and tested for purity and concentration using Nanodrop and Qubit 2.0, respectively. Libraries were prepared using KAPA, PCR-free kits. Paired-End 125 cycles sequencing was performed at the Quantitative Genomics Facility (QGF) service platform in the Department of Biosystem Science and Engineering (D-BSSE, ETH) in Basel, Switzerland, on an Illumina HiSequation 2000.

### Genome assembly and gap closing

After acquiring raw sequencing reads in the form of paired-end fastq files, we removed Illumina adapters using Trimmomatic (Bolger *et al.* 2014) and assessed read quality with FastQC (Patel and Jain 2012). FASTX toolkit was used to remove reads with Q scores < 30 (Pearson *et al.* 1997). FastQC was used once again to verify the removal of low-quality reads. Reads resulting from both isolation methods were treated independently rather than pooled. Each set of reads was mapped to the *D. magna* 2.4 reference (V2.4; Daphnia Genome Consortium) using BWAMEM (Li 2013). The resulting sam alignment file was then converted to a bam and coordinate sorted using SAMtools (Li *et al.* 2009). Reads not mapping to the *D. magna* reference were extracted using SAMtools.

To identify the optimal k-mer value for *de novo* assembly, we used KmerGenie (Chikhi and Medvedev 2014). We utilized two different *de novo* assemblers for the trimmed, unmapped paired-end reads: Velvet v.1.2.10 (Zerbino and Birney 2008) and SPAdes v.3.9.0 (Nurk *et al.* 2013). Contigs were scaffolded using the BESST scaffolding approach (Sahlin *et al.* 2014). We used GapFiller to fill in any remaining gaps in the assembly (Boetzer and Pirovano 2012).

To close the genome gap, PCR was performed using specific primers targeting the flanking region of the gap (DIV-1_GCf 5′- AGT AAC ATA GCT CAG TGG TC -3′; DIV-1_GCr 5′- ATG TTG ATT GGT GAT GCT GG-3′). The PCR reaction contained 1 µL template of DNA, 0.2 µM of each primer solution, 2 units of Phusion high-fidelity DNA polymerase (New England BioLabs), 1x Phusion HF buffer, and 200 µM deoxy-nucleotides (Promega) for a total volume of 25 µL. PCR conditions were as follows: 95° for 4 min; 35 cycles of 95 C for 30 s; 50-60° for 30 s; 72° for 2 min; and 72° for 10 min. Both negative (no DNA added) and positive controls were included in the PCR reaction. Before being used for sequencing, PCR products were purified using 1 unit of SAP shrimp alkaline phosphatase, 20 units of Exonuclease I and 1 µL of 10x SAP buffer (Fermentas). The company Microsynth (Switzerland) determined the nucleotide sequences using Sanger DNA sequencing.

The resulting assemblies were polished a final time with Pilon (Walker *et al.* 2014). LASTZ (Harris 2007) and MAFFT (Katoh and Standley 2013) were then used to evaluate individual discordances using whole genome alignment. A final consensus was generated by visualizing aligned reads with IGV (Robinson *et al.* 2011; Thorvaldsdóttir *et al.* 2013). The DIV-1 genome sequence was submitted to the European Nucleotide Archive (ENA) (http://www.ebi.ac.uk/ena) under the accession number ERP020955.

### Tests for HGT signal resulting From false positives

To test the hypothesis that several genes of DIV-1 are involved in HGT, we had to ensure that we had limited the possibility of false positive calls. As none of the DIV-1 orthologs shared very high identity with *D. magna* genes, it is unlikely that the DIV-1 genome was contaminated by host DNA as a result of misassembly. The G+C content of the candidate genes (34.8–44.1%) is similar to the G+C content of the overall DIV-1 genome (38.99%), the *D. magna* genome (33.3%) and the *D. pulex* genome (40.8%); therefore, an HGT prediction based on G+C content differences was not possible.

By using the software Daisy (Trappe *et al.* 2016) to look for split-mapping of reads from the overall datasets of both the *D. magna* and DIV-1 genomes, we were additionally able to conclude that the presence of nucleotide sequence within the DIV-1 genome did not stem from host read contamination or genome misassembly. The Daisy approach determines HGT boundaries with split-read mapping and evaluates candidate regions using read pair and coverage information (Trappe *et al.* 2016). If host read contamination is a factor, there would be significant instances of read pairs simultaneously mapped to both *D. magna* and DIV-1.

By comparing the coverages of the candidate HGT regions (Table S6), we also clarified that there was no significant difference between coverage of these regions and the genome as a whole. Furthermore, the coverage of the virus was about 8000X in both prepared libraries, while the coverage of the host genome was below 10X. For both the Daisy approach and this independent coverage analysis, we used the full read dataset rather than the read subsets, which did not initially map to the *D. magna* genome.

### Annotation and comparative genome analysis

ORFs were determined using the gene prediction software GATU, GeneMarkS v.4.28 and GLIMMER v.3.02 (Besemer *et al.* 2001; Tcherepanov *et al.* 2006; Delcher *et al.* 2007). ORFs with a methionine start codon were used for a BLASTp search against UniProtKB and UniProtKB_Viruses databases (UniProt Consortium 2015). We functionally assigned encoding proteins with e-values below 10^−3^ and identities over 20%. InterPro v.53 was used for domain and transmembrane prediction (Mitchell *et al.* 2015). In addition, we conducted a CD search against the NCBI’s conserved domain database (CDD) for predictions of conserved domains (Marchler-Bauer and Bryant 2004; Marchler-Bauer *et al.* 2015) and used SMART to explore domain architectures (Letunic *et al.* 2015). The NetNGlyc 1.0 server (http://www.cbs.dtu.dk/services/NetNGlyc/) was used for N-glycosylation prediction. Additionally, ORFs that had no homology to other proteins but contained motifs were functionally assigned. DNA repeats were identified using the program Tandem Repeats Finder v.4.09 with the parameters 2, 5 and 5 for match, mismatch and indels, respectively. The score cutoff was 40, and the maximum period size was 500 (Benson 1999). For visualization and annotation, the software tool Artemis was used (Rutherford *et al.* 2000). Collinearity analyses were done by JDotter (Brodie *et al.* 2004). DNAPlotter was used to generate the DNA map (Carver *et al.* 2009).

### Clusters of orthologs

The Proteinortho tool v.5.13 was used to detect orthologs in *Iridoviridae*, including DIV-1, and in *Iridoviridae* and *Daphnia* based on reciprocal best hits strategy (Lechner *et al.* 2011). For the analyses, all available iridoviral genome sequences (n = 53) were downloaded from the NCBI Viral Genomes Resource (Brister *et al.* 2015); additionally, re-annotated iridoviral genomes (n = 15) were downloaded from the Viral Bioinformatics Resource Center (VBRC) (Upton *et al.* 2003; Eaton *et al.* 2007). Protein sequences of the *D. magna* genome reference clone Xinb3 (from a Finnish rockpool) (GCA_001632505.1) and *D. pulex* (GCA_000187875.1) were downloaded from GenBank. Proteinortho was run using four different cutoff settings: minimal coverage 20%, minimal identity 20%, minimal connectivity 5%; minimal coverage 20%, minimal identity 20%, minimal connectivity 10%; minimal coverage 30%, minimal identity 30%, minimal connectivity 10%; and minimal coverage 30%, minimal identity 30%, minimal connectivity 5%. When the algebraic connectivity was below 10% (0.1) and 5% (0.05), respectively, Proteinortho split each group into two more dense subgroups. A connectivity of 1 indicates a perfect dense cluster of similar proteins. For the most conserved protein clusters of all *Iridoviridae*, we manually checked the clustering analyses for single missed genes and for any protein clusters that showed gaps of two or less invertebrate iridoviral proteins. We performed a tBlastn search against the nucleotide collection database in GenBank and the NCBI genomic reference sequences database to close the potential gaps. To confirm our findings, we manually inspected clusters of orthologs in *Iridoviridae* and *Daphnia* and performed a BLASTp search against the nr database in GenBank for the candidate proteins (only present in *Daphnia*, mostly absent in other *Iridoviridae*). The frog virus 3 strain RUK13 (KJ538546), the Chinese giant salamander iridovirus (KC243313), SDDV (NC_027778) and the large yellow croaker iridovirus (AY779031 and its re-annotated version LYCIV-Unk) were excluded from further analysis. The Venn diagram was calculated using R (R Development Core Team 2014), but excluded partial and partially annotated genome sequences, resulting in 49 genomes from NCBI and 14 re-annotated genomes from VBRC.

### Phylogenetic analysis

We established ten databases for annotated proteins (Table S1) with representative sequences from the virus families *Iridoviridae*, *Ascoviridae* and *Marseilleviridae* (outgroup) downloaded from GenBank. The sequences were aligned using the meta-multiple sequence alignment tool M-Coffee (Moretti *et al.* 2007). Conserved blocks were determined with the program GBlocks version 0.91b, using parameter options that allowed for smaller final blocks, gap positions within the final blocks, and less strict flanking positions using the Phylogeny.fr platform (http://www.phylogeny.fr) (Talavera and Castresana 2007; Dereeper *et al.* 2008). Protein alignments were concatenated, and phylogenetic trees were calculated using MrBayes 3.2.3 implemented in the Phylogeny.fr platform (http://www.phylogeny.fr). The maximum likelihood, maximum parsimony and distance matrix methods were implemented in MEGA6 (Tamura *et al.* 2013).

### Data Availability

The DIV-1 genome sequence has been submitted to the European Nucleotide Archive (ENA) (http://www.ebi.ac.uk/ena) under the accession number ERP020955.

## Results and discussion

### Infection trials

**Figure 2:**
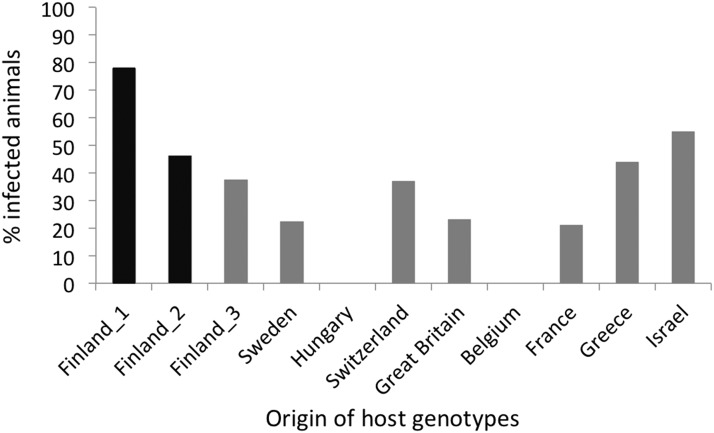
Percentage of infected replicates of 11 *D. magna* genotypes (clones) by the Daphnia iridescent virus 1 (DIV-1). The two host genotypes originating from the same site as the virus isolate are shown in black.

Of the 202 animals that were exposed to the parasite, 175 survived until day 15. After day 15, host mortality increased markedly, leading us to focus our infection data analysis on day 15. It was not possible to determine if the animals that died before day 15 were infected. In total, 60 of animals exposed to the virus became infected, while all controls remained uninfected. There was strong variation for infection among host genotypes, ranging from total resistance (0% infection) to 78% infected (Figure 2; logistic regression: *P* = 3.69e-07). The two Finnish clones that originated from the same site as the virus (Finland_1, Finland_2) showed high infection rates (46% and 78%, Figure 2). These infection trials demonstrated that WFCD is most likely caused by a virus, not by a bacterium because it is unlikely that bacteria would pass through 0.2 µm filters. The trials further show that host genotypes differ strongly in their susceptibility to virus infection.

### Ultrastructure analysis revealed icosahedral virions in the cytoplasm of infected *Daphnia magna*

**Figure 3:**
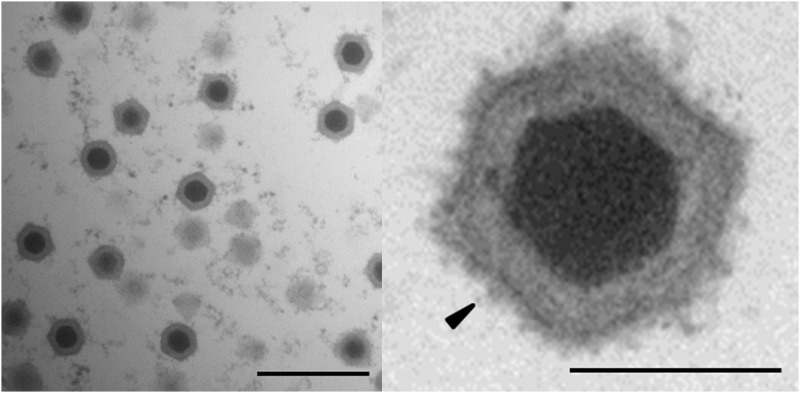
Electron micrograph of virions in a diseased *D. magna*. Ultra-thin section of a diseased *Daphnia* reveals icosahedral virions consisting of an electron-dense core and an icosahedral outer structure in the host cytoplasm. High magnification of an icosahedral virion (right) reveals that the outer surface of the viral capsid is covered with fibers (black arrow). Left: bar represents 500 nm; right: bar represents 100 nm.

No bacterial-like structures were detected in the fat cells of infected *D. magna* when we stained them with DAPI and examined them using a fluorescence microscope. However, ultrastructure analysis using an EM revealed highly abundant icosahedral virions in the cytoplasm of multiple types of tissue in the infected animals (Figure 3). Paracrystalline arrays of virions were not observed (Figure 3). The virions consisted of an electron-dense core surrounded by an icosahedral structure. They were similar to the virions Bergoin observed in *D. magna* from Mediterranean salt-marshes (Bergoin *et al.* 1984), as well as those Vávra *et al.* observed in *D. curvirostris* from Czech Republic (Vávra *et al.* 2016) and those observed in hosts infected with viruses of the family *Iridoviridae* (Jancovich *et al.* 2012). The mean particle sizes were 157 nm (n = 50) edge-to-edge and 175 nm point-to-point (measurement according to the icosahedral virus model of Mattern (1969)). The virions in daphnids that exhibited WFCD symptoms were larger than those found in *S. expinosus* (136 nm edge-to-edge and 154 nm point-to-point), but smaller than the virions observed in *D. magna* from the Mediterranean salt-marshes (180 nm) and in *D. curvirostris* from the Czech Republic (about 243 nm) (Federici and Hazard 1975; Bergoin *et al.* 1984; Vávra *et al.* 2016). Nevertheless, the EM analysis corroborates the hypothesis that the causative agent of WFCD is a virus belonging to the *Iridoviridae*.

### Assembly and gap closing of the *Daphnia* virus genome

Two Illumina libraries using different approaches for obtaining genomic DNA were treated independently and mapped to the *D. magna* 2.4 reference genome. For both read libraries, the total quantity of reads mapping to the *D. magna* reference was less than 5% of the total read dataset. For *de novo* assembly, KmerGenie suggested an optimal k-mer value of 123 for each read dataset (Chikhi and Medvedev 2014). Both Velvet (Zerbino and Birney 2008) and SPAdes (Nurk *et al.* 2013) assembly yielded two major contigs that were successfully scaffolded into a single scaffold each. After the application to fill in the gaps in the assembly, a single gap remained resulting from a region of tandem duplication. PCR and Sanger sequencing were used to close this gap and to finalize the assembly. The resulting viral genome sequence was 288,858 bp long, and the coverage of the genome combining both paired-end read datasets was ∼16,000X. The genome sequence was then taken for gene prediction and annotation.

### Daphnia iridescent virus 1 belongs to the family *Iridoviridae*

**Figure 4:**
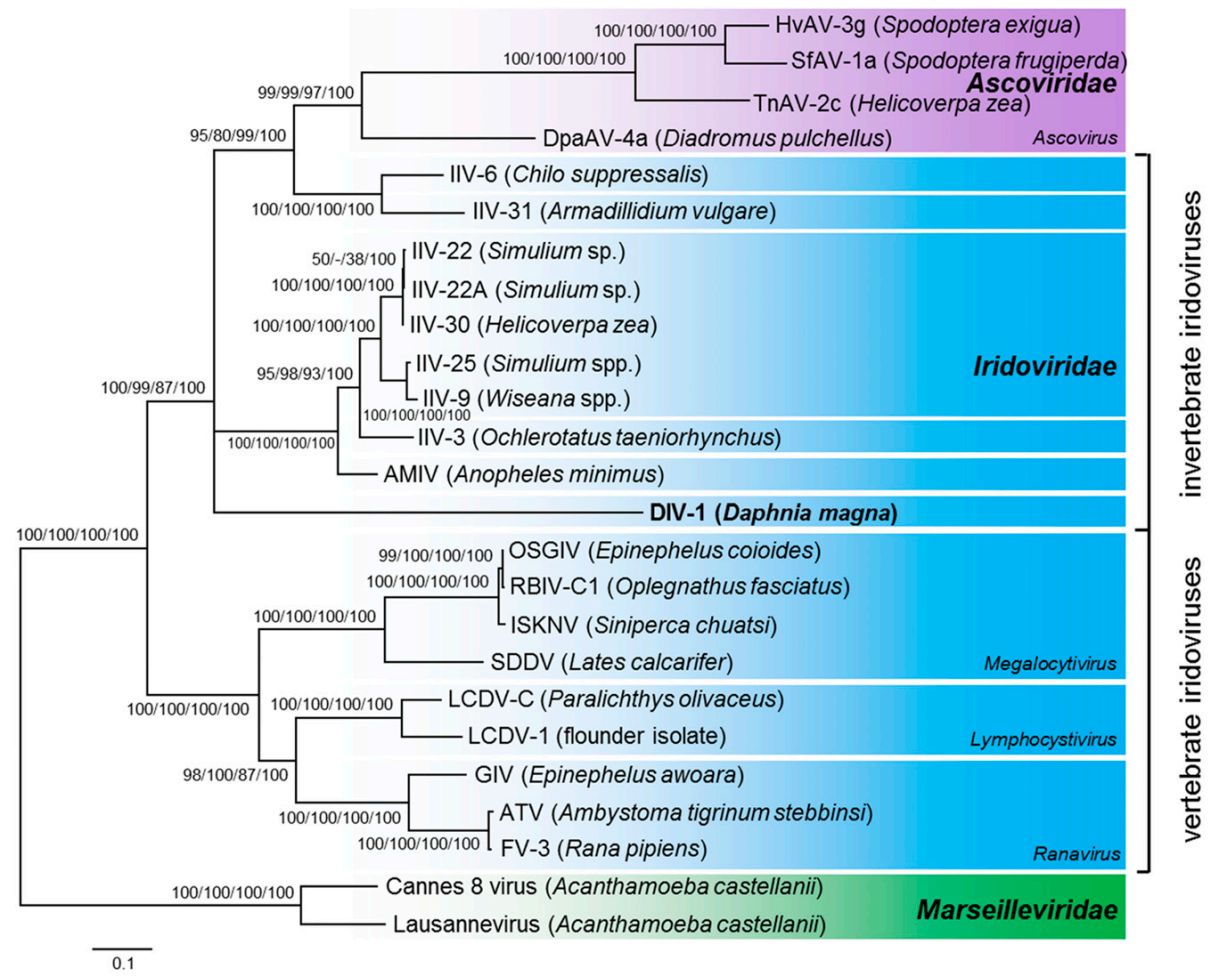
Phylogenetic position of the Daphnia iridescent virus 1 using a maximum likelihood tree based on ten concatenated proteins. Maximum likelihood, neighbor joining and maximum parsimony bootstrap values (1000 replicates), and Bayesian posterior probabilities are indicated at the inner nodes. Select members of the *Marseilleviridae* were used as the outgroup. Hosts are given in brackets. GenBank/EMBL/DDBJ accession numbers are given in Table S1.

To trace the phylogenetic relationship of the *Daphnia* virus to other documented iridoviruses (IVs), we used a previously published set of ten annotated proteins of representative members of the *Iridoviridae*, *Ascoviridae* and *Marseilleviridae* (Piégu *et al.* 2015) (Table S1). Our analysis showed that the *Iridoviridae* split into two well-supported clades: the invertebrate iridoviruses (IIVs), to which the *Daphnia* virus clustered, and the vertebrate iridoviruses (VIVs) (Figure 4). The *Ascoviridae* form the sister clade to the IIV, making the *Iridoviridae* a paraphyletic group. The *Daphnia* virus and the other crustacean IV (IIV-31) did not cluster closely. The branching order near the root of the invertebrate *Iridoviridae*/ *Ascoviridae* could not be resolved and differed between trees obtained with maximum likelihood, neighbor joining, maximum parsimony methods, and Bayesian inference. Thus, the precise phylogenetic position of the *Daphnia* virus within the invertebrate *Iridoviridae* clade remains unresolved. Given that its sequence similarity to known *Iridoviridae* is low, the *Daphnia* virus represents a novel species within the *Iridoviridae* family’s clade of IIVs. We thus propose tentatively classifying this virus as Daphnia iridescent virus 1 (DIV-1), with “*Daphnia*” indicating the host, *D. magna*, and “iridescent” referring to the iridescent shine of heavily infected daphnids. Taken together, the phylogenetic relationship of DIV-1 is consistent with the EM analysis, which shows highly abundant icosahedral virions morphologically similar to members of the *Iridoviridae*. Strong evidence indicates that the causative agent of WFCD is a virus belonging to the family *Iridoviridae*, and, therefore, that the two diseases with similar symptoms described by Bergoin *et al.* (1984) and Green (1957) are likely caused by the same pathogen species.

### Genome features of DIV-1

**Figure 5:**
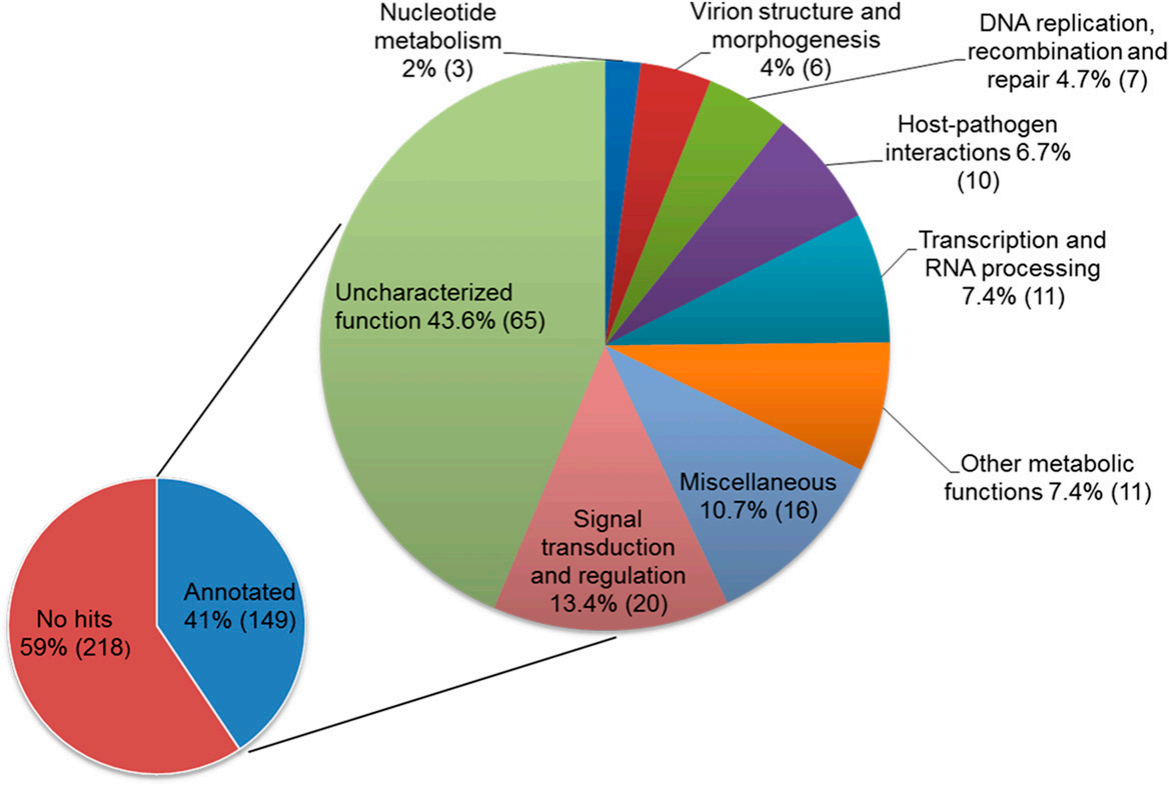
Classification of the predicted ORFs of the DIV-1 genome into functional groups. Number of ORFs within a functional group is given in brackets.

The genome features of DIV-1 are compared to other representative members of the *Iridoviridae* in Table 1. The DIV-1 genome is 288,858 bp long and contains 367 predicted open reading frames (ORFs) (Table S2 and Figure S1). 149 out of 367 ORFs are homologous to known proteins and are categorized into nine groups based on their predicted functions (Figure 5, Table S2 and Supplemental Information S1 in File S1). The remaining 218 ORFs encode hypothetical proteins with no assigned function or predicted motif (Table S2). The predicted amino acid (aa) lengths of these hypothetical proteins is 37 to 625 (average 134.9), whereas proteins with evidence for homology range from 40 to 4019 aa (average 380.1). The shorter length of the hypothetical proteins is not surprising, as homology is easier to detect for longer genes. Nevertheless, downstream analysis is necessary to verify the true nature of these predicted open reading frames.

**Table 1:** General genomic features of representative *Iridoviridae* species*^a^*

	**Invertebrate Iridoviruses**			**Vertebrate Iridoviruses**		
		***Chloriridovirus***	***Iridovirus***	***Ranavirus***	***Megalocytivirus***	***Lymphocystivirus***
**Genus**	Daphnia iridescent virus 1 (DIV-1)^b^	Invertebrate iridescent virus 3 (IIV-3, MIV)^c^	Invertebrate iridescent virus 6 (IIV-6, CIV)^d^	Frog virus 3 (FV-3)^e^	Infectious spleen and kidney necrosis virus (ISKNV)^f^	Lymphocystis disease virus 1 (LCDV-1)^g^
**Accession number**	PRJEB18974	NC_008187	NC_003038	NC_005946	NC_003494	NC_001824
**Genome size (bp)**	288,858	191,100	212,482	105,903	111,362	102,653
**Predicted ORF**	367	126^h^/126^i^	468^h^/211^i^	99^h^/97^i^	125^h^/117^i^	110^h^/108^i^
**G + C content (%)**	38.8	47.9	28.6	55.1	54.8	29.1
**Coding density^k^ (%)**	89.7	68.2/68.2	110.1/88.5	80.8/80.6	93.7/93.3	92.6/88.5
**Coding density per kbp^k^**	1.27	0.659/0.659	2.202/0.983	0.934/0.925	1.122/1.032	1.071/0.964
**Average ORF length^k^ (bp)**	706	1035/1035	500/899	864/871	835/904	864/918

a Type species according to the International Committee on Taxonomy of Viruses (http://www.ictvonline.org/); ^b^This study; ^c^(Delhon *et al.* 2006); ^d^(Jakob *et al.* 2001); ^e^(Tan *et al.* 2004); ^f^(He *et al.* 2001); ^g^(Tidona and Darai 1997); ^h^Annotated genomes available via GenBank; ^i^(Eaton *et al.* 2007); ^k^Measured by the software tool Artemis (Rutherford *et al.* 2000).

Figure S1 shows the genome map, providing a visual image of the overlapping ORF. Using the position data in Table S2, we found 62 clusters of overlapping genes with mostly two, but sometimes three, four, five or six genes involved. Of the 367 predicted genes, 139 overlap with at least one other gene.

**Figure 6:**
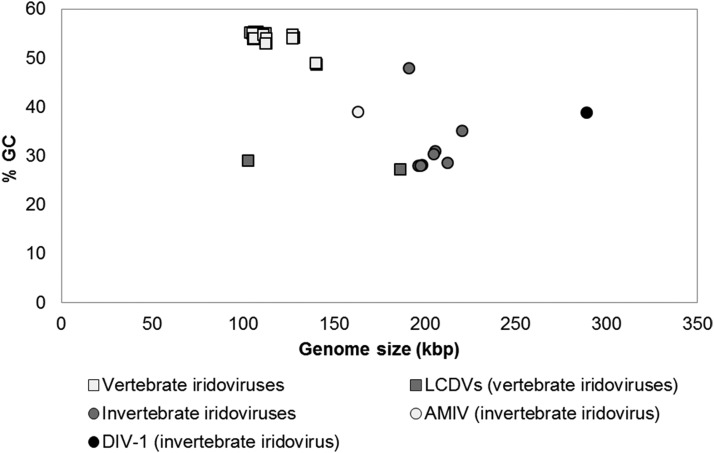
G+C content plotted against genome size in the *Iridoviridae*. In contrast to the smaller genomes of most VIVs (squares), the genomes of the IIVs are larger (circles), with DIV-1 representing the largest genome among all sequenced IIVs (black circle). Anopheles minimus irodovirus (AMIV) represents the smallest genome among IIVs (light gray circle). The G+C content of VIVs tends to be higher than that of IIVs. The two lymphocystiviruses (LCDVs) represent the VIVs with the lowest G+C content (gray).

A noticeable feature of the DIV-1 genome is its larger size compared to other *Iridoviridae* (Table 1, Figure 6). Previously sequenced IIV genomes are 163-220 kbp, while those of VIVs are 102-187 kbp. The G+C content of DIV-1 (38.8%) falls about in the middle of the reported range for other IVs (27.2 to 55.4%). Consistent with the finding that only very closely related IV show collinearity (collinear arrangement of orthologous genes) in their genomes (Eaton *et al.* 2007), the DIV-1 genome shows no collinearity with other *Iridoviridae* (IIV-3, IIV-6, FV-3, ISKNV, LCDV-1, IIV-31; Table 1), nor with the type species (SfAV-1a) of *Ascoviridae*. A total of 369 tandem repeats (TRs) are found in the DIV-1 genome: eight microsatellites (1-6 bp repeat unit size), 86 minisatellites (6-12 bp repeat unit size), and 275 longer satellite DNA (repeat units >12 bp) (Figure S1 and Table S3). Similar to other iridovirus genomes, microsatellites are rare, whereas minisatellites are found more extensively (Eaton *et al.* 2010). The TRs are located in coding as well as in non-coding regions. While the current function of TRs in *Iridoviridae* is unknown, they have been detected in late transcription in IIV-9 (Wong *et al.* 2011) and are known in other DNA viruses to contribute to genome replication and transcription (Ahrens *et al.* 1995). Changes in repeat sequences may affect host range and virus pathogenicity (Eaton *et al.* 2010; Morrison *et al.* 2014). Repeat sequences may be useful as molecular markers for viral population genetics.

### The core genes of Iridoviruses

To compare *Iridoviridae* on a genomic level, we performed clustering analyses of orthologous protein groups using published iridoviral genome sequences and the new DIV-1 genome. A set of core genes containing 26 proteins have been proposed for *Iridoviridae* (Eaton *et al.* 2007). Our analyses showed that between six and 23 proteins are conserved among all *Iridoviridae* genomes depending on the cutoff settings (Table 2 and Table S4). The analysis with the least stringent parameters identified 22 core, previously-proposed genes and one additional gene with an uncharacterized function (DIV1_252L, protein NP_149770.1 from IIV-6 genome). Using less stringent parameters, there was no respective ortholog found in the DIV-1 genome for three of the four remaining previously proposed core genes (Table S5), suggesting that, as these genes do not form orthologous clusters containing proteins for all *Iridoviridae* genomes, they do not belong to the set of *Iridoviridae* core genes. Furthermore, the proliferating cell nuclear antigen (IIV-6, NP_149899.1; FV-3, YP_031663.1), a previously proposed core gene conceivably involved in DNA replication, was also not found in the DIV-1 genome. Using less stringent parameters, the IV proteins that were annotated as proliferating cell nuclear antigens clustered into two distinct orthologous groups: the IIVs and VIVs. These two clusters may represent protein groups with different functions, or the gene was acquired independently in the two *Iridoviridae* clades, making it questionable whether it belongs to the *Iridoviridae* core genes.

**Table 2:** Set of *Iridoviridae* core genes

**Putative function**	**IIV-6 proteins**	**DIV-1 proteins**	**Orthologous cluster***^**a**^* **(alg. conn.^e^ in %)**	**Orthologous cluster^***b***^ (alg. conn. in %)**	**Orthologous cluster^***c***^ (alg. conn. in %)**	**Orthologous cluster^***d***^ (alg. conn. in %)**
**Dynein-like beta chain**	295L (NP_149758.1)	DIV1_063L	—	—	13.5	13.5
**Ribonuclease 3**	142R (NP_149605.1)	DIV1_071R	—	—	100	100
**Helicase**	184R (NP_149647.1)	DIV1_078R	—	—	84.6	84.6
**Transcription factor**	282R (NP_149745.1)	DIV1_083L	—	—	77.3	77.3
**Myristoylated membrane protein**	118L (NP_149581.1)	DIV1_133R	—	—	100	100
**CTD phosphatase-like protein**	355R (NP_149818.1)	DIV1_134R	**16.6**	**16.6**	**100**	**100**
**Kinase**	439L (NP_149902.1)	DIV1_148R	—	—	—	9.2
**DNA-directed RNA polymerase II subunit RPB1 homolog**	176R (NP_149639.1)	DIV1_159R	—	6.4	100	100
**FAD-linked sulfhydryl oxidase**	347L (NP_149810.1)	DIV1_163L	—	—	95.4	95.4
**Immediate early protein ICP-46 homolog**	393L (NP_149856.1)	DIV1_180R	—	—	39.4	39.4
**Uncharacterized protein**	117L (NP_149580.1)	DIV1_188R	—	—	19.1	19.1
**Major capsid protein**	274L (NP_149737.1)	DIV1_197L	**100**	**100**	**100**	**100**
**Ribonucleotide-diphosphate reductase small subunit**	376L (NP_149839.1)	DIV1_205L	—	—	84.8	84.8
**Transcription elongation factor S-II-like protein**	349L (NP_149812.1)	DIV1_246R	**40.9**	**40.9**	**82.5**	**82.5**
**Uncharacterized protein**	307L (NP_149770.1)	DIV1_252L	**95.2**	**95.2**	**100**	**100**
**DNA-directed RNA polymerase II subunit RPB2 homolog**	428L (NP_149891.1)	DIV1_254R	**15.5**	**15.5**	**100**	**100**
**DNA polymerase, family B**	037L (NP_149500.1)	DIV1_260R	—	—	92.4	92.4
**A32-like packaging ATPase**	075L (NP_149538.1)	DIV1_262R	**100**	**97.1**	**100**	**100**
**Helicase**	022L (NP_149485.1)	DIV1_263R	—	—	100	100
**Thymidine kinase protein**	143R (NP_149606.1)	DIV1_308L	—	—	85.1	85.1
**RAD2-like endonuclease**	369L (NP_149832.1)	DIV1_318L	—	—	13.4	13.4
**Uncharacterized protein**	287R (NP_149750.1)	DIV1_328R	—	—	—	63
**Myristylated membrane protein**	337L (NP_149800.1)	DIV1_074R, DIV1_302R	—	9.2	41.5	41.5

Clustering analysis of orthologous groups of proteins based on RBH using four different cutoff settings.

a Clustering analysis 1 (coverage 30%, identity 30%, minimal connectivity 10%); ^b^Clustering analysis 2 (coverage 30%, identity 30%, minimal connectivity 5%); ^c^Clustering analysis 3 (coverage 20%, identity 20%, minimal connectivity 10%); ^d^Clustering analysis 4 (coverage 20%, identity 20%, minimal connectivity 5%); ^e^Algebraic connectivity.

### *Iridoviridae* pan-genome and proteins conserved among DIV-1, IIVs and VIVs

**Figure 7:**
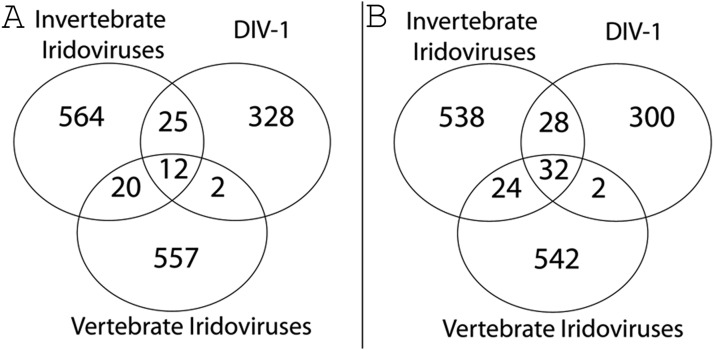
The *Iridoviridae* pan-genome and unique DIV-1 genes. Parameter settings A: minimal coverage 30%, minimal identity 30%, minimal connectivity 10%. Parameter settings B: minimal coverage 20%, minimal identity 20%, minimal connectivity 5%. The Venn diagram illustrates the *Iridoviridae* pan-genome and the number of shared and specific genes among DIV-1, the vertebrate (VIVs) and invertebrate (IIVs) iridoviruses based on ortholog clusters.

Given the phylogenetic divergence of DIV-1 from IIVs and VIVs, we were interested in those proteins that are conserved among DIV-1, IIVs and VIVs and those that are distinct only to DIV-1 and therefore might be involved in the specificity of its interaction with the *Daphnia* host. For the calculation, we relaxed assumptions about conserved genes by considering that if a particular gene were present in at least one species in a given group, it represented the entire group. The number of genes specific to the IIVs, VIVs and DIV-1 were similar in analysis using different cutoff values (Figure 7, Table S4, Table S7). Using more stringent conditions with less clustering, we found that DIV-1 shares 25 genes with the IIVs and only two genes — one involved in nucleotide metabolism (DIV1_308L) and one in protein binding (DIV1_364R) — with VIVs of the genus *Megalocytivirus* (Figure 7B), underlining the closer relationship that DIV-1 has with IIVs than VIVs. Twenty genes shared among the IIV and VIV groups were absent in DIV-1, possibly representing genes that are not necessary for infection and replication in *D. magna* hosts or genes that evolved so rapidly that assigning orthology is difficult. We found that DIV-1 contained about 300 genes (Figure 7) not found in any of the other IVs genomes. Most of these genes (214) encoded hypothetical proteins with no assigned function or predicted motif (Table S2, Table S4). The remaining 86 genes encoded proteins involved in DNA replication, recombination and repair, signal transduction and regulation, transcription and mRNA biogenesis, host-pathogen interactions, virion structure and morphogenesis, lipid metabolism, protein – protein interaction, or other, uncharacterized functions. The relatively high number of specific genes in the DIV-1 genome and the fact that its coding density is similar to other *Iridoviridae* goes hand-in-hand with DIV-1’s larger genome size (Figure 6 and Table 1).

### DIV-1 proteins shared With *Daphnia*

Horizontal gene transfer (HGT) is a well known mode by which species acquire new genes. Many of the predicted ORFs in the DIV-1 genome are either orthologs to proteins belonging to *Iridoviridae* members or homologs to other members of the proposed order Megavirales (Colson *et al.* 2013). Most of the remaining and assigned ORFs, on the other hand, are similar to genes in other viruses, phages, bacteria and eukaryotes. Because IVs are known to encode a number of proteins that are orthologs to cellular proteins (Tidona and Darai 2000), we closely investigated proteins that showed homology between DIV-1 and its *Daphnia* host. We used a clustering analysis based on reciprocal best blast search with different cutoff settings for the reported *D. magna* and *D. pulex* proteins, for all available *Iridoviridae* genomes (n = 68), and for the DIV-1 genome. After further examining the candidates, we selected for additional analysis those proteins present only in *D. magna* or in both *D. magna* and *D. pulex*, but absent in most *Iridoviridae* (Table S6). This analysis identified 22 proteins as potential candidates for HGT events between DIV-1 and its hosts or their ancestors (Table S6). These genes were related to such functions as host-virus interaction (DIV1_038L, DIV1_293R), protein–protein interactions (DIV1_003R, DIV1_065L, DIV1_082L, DIV1_142L, DIV1_270L), replication (DIV1_078R), transcription and RNA processing (DIV1_186R), signal transduction and regulation (DIV1_210L, DIV1_213L, DIV1_214L, DIV1_216L, DIV1_299L, DIV1_324R), or other metabolic (DIV1_151L, DIV1_280R, DIV1_288R) and uncharacterized functions (DIV1_035L, DIV1_59R, DIV1_098L, DIV1_337L). We will discuss three of these genes here in more detail.

ORF DIV1_038L encodes a Golgi anti-apoptotic protein (GAAP) of the BI-1-like superfamily. Located in the Golgi and the ER of the host cell, the viral GAAP acts as an apoptosis inhibitor, affecting virus virulence. It has been linked to cancer progression and metastasis and can complement the eukaryotic GAAP (Gubser *et al.* 2007; Rojas-Rivera and Hetz 2015). ORF DIV1_038L is most similar to *D. magna’s* protein lifeguard 4 (KZS20159.1, e-value 3e^-67^, ident. 51%), to a hypothetical protein of *D. pulex* (EFX68523.1, e-value 8e^-59^, ident. 45%), and to other eukaryotic proteins of the BI-1 -like superfamily. In viruses, DIV-1 GAAP has an ortholog in the megalocytiviral Scale drop disease virus (SDDV, ORF_095L (YP_009163856.1), e-value 9e^-19^, ident. 29%), but shows the highest similarity to the GAAP of the Vaccinia virus (AAV98625.1, e-value 1e^-28^, ident. 34%) and other poxviruses, members of the Megavirales. Based on our cluster analyses, the DIV-1 GAAP protein was probably acquired either independently from its eukaryotic host, or it was already present in the ancestors of the *Iridoviridae* or the Megavirales, where it was transferred from DIV-1 to its host.

Another gene, ORF DIV1_078R, encodes a putative helicase with a PriCT-2 domain, a domain of the VirE superfamily that contains virulence-associated proteins, and a SF3 helicase domain involved in DNA replication and only found in viruses or prophage remnants of cellular genomes (Iyer *et al.* 2004). Depending on the parameters used in the clustering approaches for *Daphnia* and *Iridoviridae* proteins or for *Iridoviridae* proteins only, this protein is orthologous to a *D. magna* protein and iridoviral proteins (*e.g.*, IIV-6_184R, NP_149647.1) but is not reported in *D. pulex*. Although proposed to be a core gene of IVs (Eaton *et al.* 2007), the C-terminal subunit of this protein is highly similar to an uncharacterized protein of *D. magna* (KZS16065.1, e-value 6e^-82^, identity 47%), suggesting that it may be a candidate for a relatively recent HGT event from DIV-1 to *D. magna*.

A third gene, ORF DIV1_142L, encodes a von Willebrand factor A (vWFA) domain-containing protein with a VIT domain and a vWFA domain, and is probably involved in protein–protein interaction, in protease inhibition, and in extracellular matrix binding and/or stability (Whittaker and Hynes 2002). Proteins containing the vWA domain are present in eukaryotes, bacteria, archaea, phages, and viruses such as NCLDVs (Whittaker and Hynes 2002; Schroeder *et al.* 2009). This protein is orthologous and highly similar to an uncharacterized protein of *D. magna* (KZS17173.1, e-value 2e^-106^, ident. 32%) and a hypothetical protein of *D. pulex* (EFX90299.1, e-value 9e^-99^, ident. 30%), but no ortholog is found in other *Iridoviridae*, suggesting that DIV-1 might have acquired this protein by HGT from *D. magna*.

### Orthologs shared by invertebrate iridoviruses

Between 11 and 19 protein clusters are shared by all invertebrate iridoviruses, depending on the cutoff parameter used (Table 3 and Table S4). Six protein clusters (DIV1_086L, DIV1_162R, DIV1_166R, DIV1_219L, DIV1_242L, and DIV1_290L) are consistent in all four analyses containing a metallopeptidase (DIV1_162R) that is conceivably involved in host-pathogen interaction or that acts on the extracellular matrix, facilitating virus spread within the host. There is also a high mobility group protein homolog (DIV1_242L) that may play a role in genome conformation, and four uncharacterized proteins (DIV1_086L, DIV1_166R, DIV1_219R, DIV1_290L) (Delhon *et al.* 2006). These IIV-specific orthologs may represent genes needed for infection and spread in an invertebrate host.

**Table 3:** Conserved proteins among all IIVs

**Putative function**	**IIV-6 proteins**	**DIV-1 proteins**	**Orthologous cluster***^a^* **(alg. conn.^e^ in %)**	**Orthologous cluster^b^ (alg. conn. in %)**	**Orthologous cluster^c^ (alg. conn. in %)**	**Orthologous cluster^d^ (alg. conn. in %)**
**Metallopeptidase**	165R (NP_149628.1)	DIV1_064R	18.2	18.2	—	—
**Metallopeptidase/ metalloprotease**	165R (NP_149628.1)	DIV1_064R, DIV1_301L	—	—	16.7	16.7
**Uncharacterized protein**	342R (NP_149805.1)	DIV1_086L	**100**	**100**	**100**	**100**
**Kinase**	439L (NP_149902.1)	DIV1_148R	54.6	54.6	—	—
**Uncharacterized protein**	155L (NP_149618.1), 149L (NP_149612.1)	DIV1_157R	—	—	—	6.7
**DNA-directed RNA polymerase II subunit RPB1 homolog**	176R (NP_149639.1)	DIV1_159R	100	—	—	—
**Metallopeptidase**	136R (NP_149599.1)	DIV1_162R	**100**	**100**	**100**	**100**
**Uncharacterized protein**	309L (NP_149772.1)	DIV1_165R	—	—	41.7	41.7
**Uncharacterized protein**	415R (NP_149878.1)	DIV1_166R	**66.7**	**66.7**	**66.7**	**66.7**
**Immediate early protein ICP-46 homolog**	393L (NP_149856.1)	DIV1_180R	16.4	16.4	—	—
**Hypothetical protein**	357R (NP_149820.1)	DIV1_187L	—	—	—	8.3
**Uncharacterized protein**	117L (NP_149580.1)	DIV1_188R	100	—	—	—
**Serine/threonine-protein kinase**	389L (NP_149852.1)	DIV1_216L	—	—	—	8.3
**Uncharacterized protein**	395R (NP_149858.1)	DIV1_219R	**38.2**	**38.2**	**100**	**100**
**High mobility group protein homolog**	401R (NP_149864.1)	DIV1_242L	**100**	**100**	**100**	**100**
**Serine/threonine-protein kinase**	098R (NP_149561.1)	DIV1_245L	—	—	100	100
**Hypothetical protein**	385L (NP_149848.1)	DIV1_253R	—	—	—	7.3
**Uncharacterized protein**	198R (NP_149661.1)	DIV1_255L	—	—	100	100
**Uncharacterized protein**	268L (NP_149731.1)	DIV1_256R	—	—	41.7	41.7
**Uncharacterized protein**	350L (NP_149813.1)	DIV1_257R	66.7	66.7	100	—
**Zinc finger domain protein**	077L (NP_149540.1)	DIV1_261R	—	—	43.3	43.3
**Uncharacterized protein**	325L (NP_149788.1), 203L (NP_149666.1)	DIV1_272L	—	—	50	9.1
**DNA ligase**	205R (NP_149668.1)	DIV1_273L	—	—	100	100
**Uncharacterized protein**	329R (NP_149792.1)	DIV1_290L	**100**	**100**	**100**	**100**
**RAD2-like endonuclease**	369L (NP_149832.1)	DIV1_318L	57.6	57.6	—	—
**Uncharacterized protein**	378R (NP_149841.1)	DIV1_025L, DIV1_173R	—	—	—	7.6

Clustering analysis of orthologous protein groups based on RBH using four different cutoff settings.

a Clustering analysis 1 (coverage 30%, identity 30%, minimal connectivity 10%); ^b^Clustering analysis 2 (coverage 30%, identity 30%, minimal connectivity 5%); ^c^Clustering analysis 3 (coverage 20%, identity 20%, minimal connectivity 10%); ^d^Clustering analysis 4 (coverage 20%, identity 20%, minimal connectivity 5%); ^e^Algebraic connectivity.

### DIV-1 genes may play a role in RNA capping

RNA capping (in which the viral mRNA 5′ ends are capped) is an essential modification used by all eukaryotes and most viruses. Uncapped RNAs are degraded by cellular exonuclease, a process that may also trigger antiviral immune responses. Viruses that lack the cap structure either attach a VPg-like protein to their RNA 5′ end, or they recruit a eukaryotic multiprotein complex for translation initiation (Decroly *et al.* 2012; Ferron *et al.* 2012). All eukaryotic species and most DNA viruses share a three-step capping process to form the cap at the 5′ end. This process consists of the following steps: (1) hydrolysis of the γ-phosphate of the primary transcript by an RNA triphosphatase (RTPase); (2) transfer of GMP to the 5′-diphosphate RNA to form a GpppNp-RNA cap by an RNA guanylyltransferase (GTase); and (3) methylation of the cap guanine to form the m^7^GpppNp-RNA structure by a cap-specific RNA (guanine-N7) methyltransferase (MTase) (Shuman 2002). Some eukaryotic DNA viruses, such as SV40, adenovirus and herpesvirus, are known to exploit host enzymes for RNA capping. In contrast to these, many of the dsDNA viruses — poxviruses, baculoviruses, African swine fever virus, *Chlorella* virus, Coccolithovirus, and certain iridoviruses —encode some or all of the enzymes necessary for synthesis and capping of viral mRNAs. Therefore, the RNA capping machinery represents an attractive target for antiviral drugs (Decroly *et al.* 2012; Ferron *et al.* 2012).

The DIV-1 genome encodes genes that are probably involved in RNA capping. ORF DIV1_186R and DIV1_263R encode two helicases that belong to either the SF1 or SF2 helicase superfamily. They carry the classical Walker A motif forming the P-loop and the Walker B motif (D-E-X-D box) with an Mg^2+^ binding site needed for hydrolysis. These proteins with NTPase-helicase activity may function as RNA triphosphatases responsible for the first step of the cap formation (Decroly *et al.* 2012; Ferron *et al.* 2012). For the second step of capping, ORF DIV1_060L encodes an mRNA-capping enzyme containing a nucleotidyltransferase domain and an oligonucleotide-binding domain that may function as guanylyltransferase, which adds the cap structure (Decroly *et al.* 2012). Finally, ORF DIV1_348L encodes a class 1 S-adenosylmethionine-dependent methyltransferase (AdoMet-MTase). Some AdoMet-MTases contain a domain that conducts both RNA guanine-N7-methyltransferase (RNMT) activity and nucleoside-2′-O-methyltransferase activity. RNA guanine-N7-methyltransferase (RNMT) activity transfers the methyl group from S-adenosyl-L-methionine (AdoMet) to the cap guanine, while nucleoside-2′-O-methyltransferase activity transfers the methyl group from S-adenosyl-L-methionine (AdoMet) to the ribose-2’-O position of the first nucleotide of the mRNA (Decroly *et al.* 2012; Ferron *et al.* 2012). By potentially combining both activities, ORF DIV1_348L may conceivably be involved in the final step of the capping. In addition, ORF DIV1_203R encodes an RNMT-activating mini protein (RAM) consisting of an RNMT-activating domain and an RNA-binding domain. DIV-1 RAM may also interact with the AdoMet-MTase (DIV1_348L), enhancing the mRNA binding and cap methyltransferase activity, as it is known to do for other RAM (Gonatopoulos-Pournatzis *et al.* 2011). The AdoMet-MTase (DIV1_348L), RAM (DIV1_203R) and the mRNA capping enzyme (DIV1_060L) have no orthologs in other IVs, although proteins similar to the mRNA capping enzyme (DIV1_060L) have been found in the megalocytiviruses. Therefore, DIV-1 may represent the first example of an IIV that uses RNA capping, although the functions of the proteins must be further examined.

### Conclusion

The Daphnia iridescent virus 1 (DIV-1), the causative agent of White Fat Cell Disease (WFCD) in the water flea *D. magna*, is a member of the invertebrate IV clade within the virus family *Iridoviridae*. Compared to other IVs of this group, the DIV-1 genome has apparently undergone a substantial gene loss and acquired a number of unique new genes after splitting from its most recently known ancestor. Horizontal gene uptake probably helped the virus adapt to its crustacean host. DIV-1 has a unique position within the IIVs, as it encodes genes that are probably involved in RNA capping. These genes are not usually found in IIVs. The uniqueness of DIV-1 among the IVs is also seen in that it contains genes for host–pathogen interaction and pathogenicity. Furthermore, with about 300 DIV-1 specific ORFs containing characterized and hypothetical proteins, the virus is strongly diverged from other IVs. Nevertheless, all IIVs share a set of specific genes involved primarily in conserved functions and host-pathogen interactions that enable these viruses to infect and replicate in invertebrate hosts.

DIV-1 is widespread in natural populations of *D. magna* in Western Eurasia and very easy to recognize, thus making it a good prospect for ecological and epidemiological research. Furthermore, it is easy to maintain in laboratory cultures of its host, allowing unprecedented opportunities for experimental and molecular studies. As little is known about iridoviruses and *Daphnia* viruses in general, DIV-1 opens up a promising new area for research in iridovirus–host interactions and their influence on the ecosystem.

## Supplementary Material

Supplemental Material is available online at www.g3journal.org/lookup/suppl/doi:10.1534/g3.117.300429/-/DC1.
