# Supplementary material for: The End of a 60-year Riddle: Identification and Genomic Characterization of an Iridovirus, the Causative Agent of White Fat Cell Disease in Zooplankton
Source: G3 (Bethesda). 2018 Feb 27;8(4):1259–72. doi: 10.1534/g3.117.300429 (PMC5873915; doi:10.1534/g3.117.300429)
Supplement: Supplementary file 1 [file 1259FileS1.pdf]

Supplemental Information S1 to article: **The end of a 60-year riddle: Identification and genomic characterization of an iridovirus, the causative agent of White Fat Cell Disease in zooplankton**

Elena R. Toenshoff[1], Peter D. Fields[1], Yann X. Bourgeois[1,#a] and Dieter Ebert[1*]

[1] Basel University, Department of Environmental Sciences, Zoology, Vesalgasse 1, CH-4051 Basel, Switzerland

[#a] Current Address. New York University Abu Dhabi, Saadiyat Island campus, P.O. box 129188, Abu Dhabi, United Arab Emirates

Supplemental Information S1. Description of the putative functions of the DIV-1 genome

DNA replication and repair

Iridoviridae conduct a unique DNA replication cycle including a nuclear and a cytoplasmic phase (Chinchar *et al.* 2009). A putative DNA polymerase of the B family (DIV1_260R) and a putative DNA ligase (DIV1_273L) are encoded on the DIV-1 genome, which are involved in DNA replication. For DNA repair the DIV-1 genome encodes a putative DNA helicase Pif1-like (DIV1_077R) and a putative RAD2-like endonuclease (DIV1_318L). ORF DIV1_078R encodes a putative helicase which may act as primase catalyzing the synthesis of short nucleotide molecules. ORF DIV1_195R encodes a protein with a ribonuclease H-like domain that is probably involved in concatemer resolution in the host cytoplasm, DNA replication and procession, similar as the poxviral A22 protein with Holliday junction resolvase activity (Garcia *et al.* 2006). Additionally, ORF DIV1_116L encodes a nuclease with a SNase-like domain of the SNc superfamily, which may catalyze the hydrolysis of single- and double-stranded DNA and RNA. ORF DIV1_116L has no orthologue in other IVs, but is present in other Megavirales such as *Phycodnaviridae*, therefore this gene may get lost in other IVs or was acquired by HGT for the adaptation of DIV-1 to the specific life cycle within *D. magna*.

Nucleotide metabolism

The DIV-1 genome contains protein coding genes for nucleotide transport and metabolism such as the small and large subunits of the ribonucleotide-diphosphate reductase (DIV1_205L and DIV1_321R), a key enzyme catalyzing the synthesis of

deoxyribonucleotides in the cytoplasm, and a thymidine kinase (DIV1_308L), which was already present in the Megavirales and an ancestor of IVs (Iyer *et al.* 2006). An UTPase, important for regulation of dUTP levels, was not found in the DIV-1 genome, similar as for the invertebrate iridovirus IIV-3, lymphocystiviruses and megalocytiviruses. To challenge the host nucleotide precursor pool for viral DNA synthesis seems an ancient strategy of DNA viruses, which might trigger the gene loss of nucleotide metabolism enzymes (Iyer *et al.* 2006).

Transcription and mRNA biogenesis

Transcription occurs in three coordinated phases, the immediate early (IE) and delayed early (DE) gene expression in the nucleus using viral DNA as template and catalyzed by host RNA polymerase II, and the late (L) transcripts in the cytoplasm catalyzed by a virus-encoded RNA polymerase II (Williams *et al.* 2005). In DIV-1, proteins that are related to DNA transcription are the two large subunits of DNA-dependent RNA polymerase II (DIV1_159R and DIV1_254R), which is found in all IVs and most likely existed already in an ancestor of the Megavirales (Iyer *et al.* 2006). ORF DIV1_083L encodes a transcription factor belonging to the poxvirus VLTF3-like family of transcription factors, which regulates the late transcription. This gene was found in most IVs. ORF DIV1_071R encodes a putative ribonuclease 3 which is involved in RNA binding and processing and conserved in IVs. In addition, ORF DIV1_246R encodes the transcription elongation factor S-II-like protein (TFIIS), which is involved in binding to RNA polymerases, transcript elongation and activation of nascent RNA cleavage of the RNA polymerase II. This transcription factor is present in almost all IVs and seems to be already encoded by an ancestor of the Megavirales (Iyer *et al.* 2006). ORF DIV1_147L encodes the RNA polymerase subunit RPB5, which is a shared subunit of RNA polymerases and plays a role in transcription activation (Miyao et al, 1998). This gene is found in most IVs and the Megavirales (Iyer *et al.* 2006).

Virus structure and morphogenesis

Iridovirus particles consist of an inner DNA/ protein core surrounded by a lipid membrane with transmembrane proteins connecting the lipid membrane with the surrounding capsid shell (Chinchar *et al.* 1984). ORF DIV1_197L encodes a putative major capsid protein (MCP), which is a major component of the virus capsid and necessary for virion assembly (Chinchar *et al.* 1984; Sample *et al.* 2007). The MCP represents a core gene of *Iridoviridae*. Various membrane proteins were found in the DIV-1 genome such as ORF DIV1_200L encoding a putative membrane protein, ORF DIV1_074R and ORF DIV1_302R, two

paralogues, encoding myristylated membrane proteins with unknown function, which belong to the set of core genes of *Iridoviridae*. Further, ORF DIV1_133R encodes a myristoylated membrane protein, a lipid membrane protein of the L1R_F9L super family also found in members of the Megavirales. This protein may have an essential role in virion assembly, similar to the orthologue ORF53R (YP_031631.1) of FV3. After blocking of ORF53R expression FV3 showed a reduced virus titer and an increase of not encapsulated protein-DNA complexes within the host (Whitley *et al.* 2010). Moreover, ORF DIV1_262R encodes a putative A32-like packaging ATPase of the P-loop NTPase superfamily, found in other IVs and members of the Megavirales. For instance, the A32 protein of the vaccinia virus (*Poxviridae*) is involved in ATP-consuming packaging of the DNA in virus particles (Koonin *et al.* 1993; Cassetti *et al.* 1998), therefore DIV1_262R is probably involved in DNA packaging.

DIV-1 genes that may be involved in host-pathogen interactions

The DIV-1 genome contains genes, either unique to itself, or shared with other IVs, that purportedly mediate host-pathogen interactions. For instance, two genes, similar to other IVs, are inhibitors of apoptosis: ORF DIV1_038L encodes a Golgi anti-apoptotic protein (GAAP) probably acquired by HGT (as mentioned above), and ORF DIV1_056R encodes a putative apoptosis inhibitor protein (IAP) containing a BIR domain. In IIV-6, IAP is transcribed early on in the infection and inhibits apoptosis by blocking an initiator caspase necessary for cell death in *Drosophila* (İnce *et al.* 2008; Williams *et al.* 2009). However, because the IIV-6 IAP encodes a C-terminal RING finger motif that is absent in the DIV-1 IAP, the function of the DIV-1 IAP requires further exploration. In addition, four proteinases (DIV1_064R, DIV1_089L, DIV1_162R, DIV1_301L), also found in other IVs, are encoded on the DIV-1 genome and are likely involved in proteolytic activities and host interactions. For instance, ORF DIV1_064R encodes a Zinc-dependent metallopeptidase with a signal peptide, which is conceivably translocated to the extracellular matrix to facilitate virus spread in the host or to develop immune responses (Delhon *et al.* 2006). ORF DIV1_089L encodes a papain-like proteinase of the C1 peptidase family. In general, cysteine peptidases are a diverse protein family found in most organisms, and they play a central role in pathogenicity and parasite biology (Atkinson *et al.* 2009; Novinec and Lenarčič 2013). This protein has orthologues in other IIVs and in members of the Megavirales but is not found in VIVs except lymphocystiviruses; therefore, we assume the papain-like proteinase is lost in VIVs of the *Megalocytivirus* and *Ranavirus* genera. Furthermore, ORF DIV1_293R encodes an uncharacterized protein containing four immunoglobulin (Ig) domains of the subtype I set-C2 and a signal peptide at the N-terminus. Because this protein is well glycosylated with neither a transmembrane

segment nor a glycosylphosphatidylinositol (GPI) anchor site, it is most likely present in soluble form in *Daphnia*. This protein has no orthologue in other IVs, but proteins with Ig domains involved in viral replication, cytoskeleton reorganization and immune response regulation are known for the Singapore grouper iridovirus (SGIV) and Grouper iridovirus (GIV) (Song *et al.* 2004; Tsai *et al.* 2005; Yan *et al.* 2014). However, the soluble alpha/beta interferon (IFN) receptor (B18R) with three Ig domains of the Vaccinia virus (poxvirus) binds to the surface of infected and uninfected cells and protects cells from the antiviral effect of IFN (Alcamí *et al.* 2000). Therefore, DIV1_293R could also be involved in effector molecule binding of the *Daphnia* immune system. ORF DIV1_184L encodes a putative membrane glycoprotein with no orthologue in IVs, although it is similar to the glycoprotein gp2 of the dsDNA equine herpesvirus 1 (ORF71 (AAK61484.1), e-value 1.4 x 10^{-5}, ident. 46.6%). The herpes viral glycoprotein gp2 is located on the surface of virion envelopes and infected cell membranes (McGeoch *et al.* 1993; Marshall *et al.* 1997), and Gp2 mutants have been shown to be less virulent, exhibiting reduced growth in lungs of mice (Sun *et al.* 1994). Therefore, DIV1_184L might also be involved in pathogenicity. ORF DIV1_244L encodes a FAS1 domain-containing protein of the fasciclin superfamily that is present in many secreted and membrane-anchored proteins. This domain is also found in proteins (no orthologues) of all IIVs and is probably important in interactions with invertebrate hosts. Finally, ORF DIV1_270L encodes a putative DnaJ-like protein of the DnaJ superfamily, classified as a type III protein containing a J-domain with a HPD motif for Hsp70 interaction. DnaJ-like proteins are found in Megavirales, but there is no orthologue in IVs. In general, heat shock protein 40 (Hsp40/ DnaJ) is involved in the virus life cycle and virus pathogenicity in a wide range of viral infections (Knox *et al.* 2011). Therefore, DIV1_270L might play a role in host-pathogen interaction and infection.

Supplemental references

Alcamí, A., Symons, J.A., Smith, G.L., 2000 The vaccinia virus soluble alpha/ beta interferon (IFN) receptor binds to the cell surface and protects cells from the antiviral effects of IFN. *J Virol* **74**:11230-11239.

Atkinson, H.J., Babbitt, P.C., Sajid, M., 2009 The global cysteine peptidase landscape in parasites. *Trends Parasitol* **25**:573-581.

Cassetti, M.C., Merchlinsky, M., Wolffe, E.J., Weisberg, A.S., Moss, B., 1998 DNA packaging mutant: repression of the vaccinia virus A32 gene results in noninfectious, DNA-deficient, spherical, enveloped particles. *J. Virology* **72**:5769-5780.

Chinchar, G.V., Goorha, R., Granoff, A., 1984 Early proteins are required for the formation of frog virus 3 assembly sites. *Virology* **135**:148-156.

Chinchar, V.G., Hyatt, A., Miyazaki, T., Williams, T., 2009 Family *Iridoviridae*: poor viral relations no longer, pp. 123-170 in *Lesser known large dsDNA viruses*, edited by J.L. Van Etten. Springer, Berlin, Heidelberg.

Delhon, G., Tulman, E.R., Afonso, C.L., Lu, Z., Becnel, J.J. et al.,, 2006 Genome of invertebrate Iridescent virus type 3 (mosquito iridescent virus). *J Virol* **80**:8439-8449.

Garcia, A.D., Otero, J., Lebowitz, J., Schuck, P., Moss, B., 2006 Quaternary structure and cleavage specificity of a poxvirus holliday junction resolvase. *J Biol Chem* **281**:11618-11626.

İnce, İ.A., Westenberg, M., Vlak, J.M., Demirbağ, Z., Nalçacıoğlu, R. et al.,, 2008 Open reading frame 193R of Chilo iridescent virus encodes a functional inhibitor of apoptosis (IAP). *Virology* **376**:124-131.

Iyer, L.M., Balaji, S., Koonin, E.V., Aravind, L., 2006 Evolutionary genomics of nucleo-cytoplasmic large DNA viruses. *Virus Research* **117**:156-184.

Knox, C., Luke, G.A., Blatch, G.L., Pesce, E.-R., 2011 Heat shock protein 40 (Hsp40) plays a key role in the virus life cycle. *Virus Res* **160**:15-24.

Koonin, E.V., Senkevich, T.G., Chernos, V.I., 1993 Gene A32 product of vaccinia virus may be an ATPase involved in viral DNA packaging as indicated by sequence comparisons with other putative viral ATPases. *Virus Genes* **7**:89-94.

Marshall, K.R., Sun, Y., Brown, S.M., Field, H.J., 1997 An equine herpesvirus-1 gene 71 deletant is attenuated and elicits a protective immune response in mice. *Virology* **231**:20-27.

McGeoch, D.J., Barnett, B.C., MacLean, C.A., 1993 Emerging functions of alphaherpesvirus genes. *Semin Virol* **4**:125-134.

Novinec, M., Lenarčič, B., 2013 Papain-like peptidases: structure, function, and evolution. *bmc* **4**:287-308.

Sample, R., Bryan, L., Long, S., Majji, S., Hoskins, G. et al.,, 2007 Inhibition of iridovirus protein synthesis and virus replication by antisense morpholino oligonucleotides targeted to the major capsid protein, the 18 kDa immediate-early protein, and a viral homolog of RNA polymerase II. *Virology* **358**:311-320.

Song, W.J., Qin, Q.W., Qiu, J., Huang, C.H., Wang, F. et al.,, 2004 Functional genomics analysis of Singapore grouper iridovirus: complete sequence determination and proteomic analysis. *J Virol* **78**:12576-12590.

Sun, Y., MacLean, A.R., Dargan, D., Brown, S.M., 1994 Identification and characterization of the protein product of gene 71 in equine herpesvirus 1. *J Gen Virol* **75**:3117-3126.

Tsai, C.-T., Ting, J.-W., Wu, M.-H., Wu, M.-F., Guo, I.-C. et al.,, 2005 Complete genome sequence of the Grouper iridovirus and comparison of genomic organization with those of other iridoviruses. *J Virol* **79**:2010-2023.

Whitley, D.S., Yu, K., Sample, R.C., Sinning, A., Henegar, J. et al.,, 2010 Frog virus 3 ORF 53R, a putative myristoylated membrane protein, is essential for virus replication in vitro. *Virology* **405**:448-456.

Williams, T., Barbosa-Solomieu, V., Chinchar, V.G., 2005 A decade of advances in iridovirus research. *Adv Virus Res* **65**:173-248.

Williams, T., Chitnis, N.S., Bilimoria, S.L., 2009 Invertebrate iridovirus modulation of apoptosis. *Virol Sin* **24**:295-304.

Yan, Y., Cui, H., Guo, C., Wei, J., Huang, Y. et al.,, 2014 Singapore grouper iridovirus-encoded semaphorin homologue (SGIV-sema) contributes to viral replication, cytoskeleton reorganization and inhibition of cellular immune responses. *J Gen Virol* **95**:1144-1155.
