# Supplementary material for: The End of a 60-year Riddle: Identification and Genomic Characterization of an Iridovirus, the Causative Agent of White Fat Cell Disease in Zooplankton
Source: G3 (Bethesda). 2018 Feb 27;8(4):1259–72. doi: 10.1534/g3.117.300429 (PMC5873915; doi:10.1534/g3.117.300429)
Supplement: Supplementary file 2 [file 1259FigureS1.pdf]

Supplemental Fig. S1. Genome map of Daphnia iridescent virus 1 (~288 kbp). Based on Blastp search, ORFs homolog to *Iridoviridae* (n = 51) are displayed in dark blue, homologs to *Daphnia* (n = 15) in red, all other ORFs are indicated in light green (Supplemental Table S2). Tandem repeats (TRs, n = 369) are indicated in dark green. Deviations from the average G+C content (38.8 %) are displayed in grey and black, respectively. Scale bar in bp.

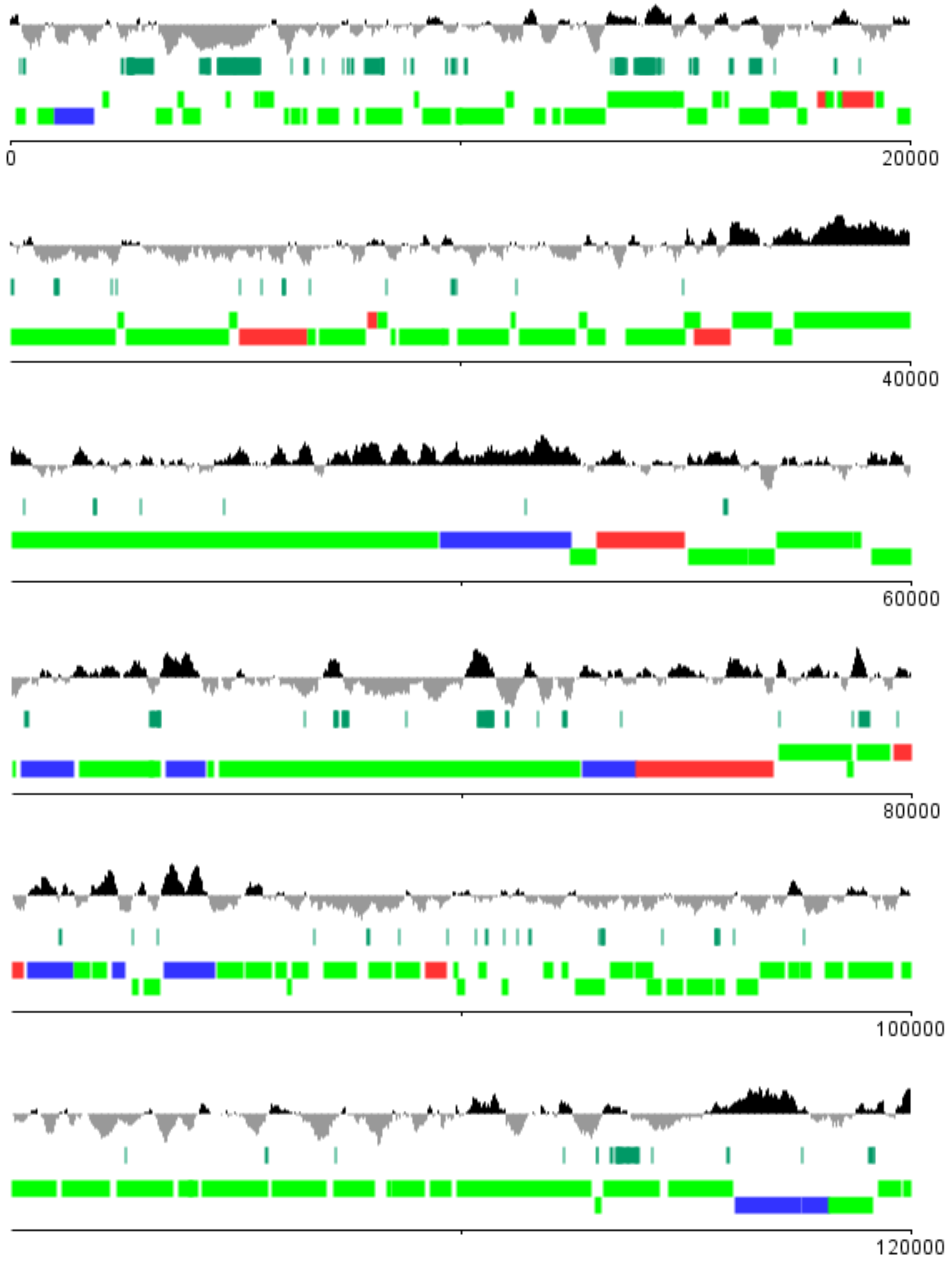

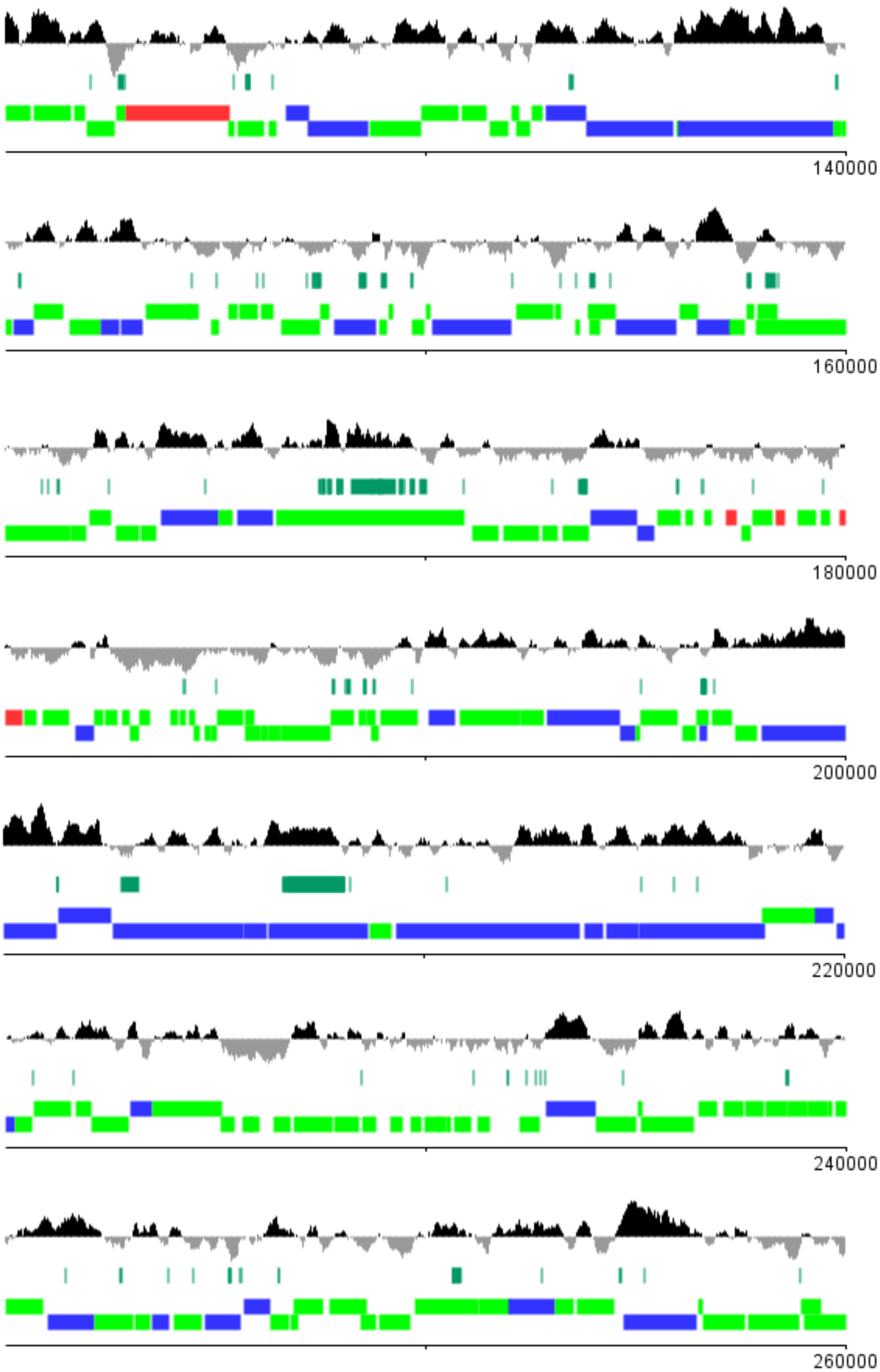
140000
160000
180000
200000
220000
240000
260000

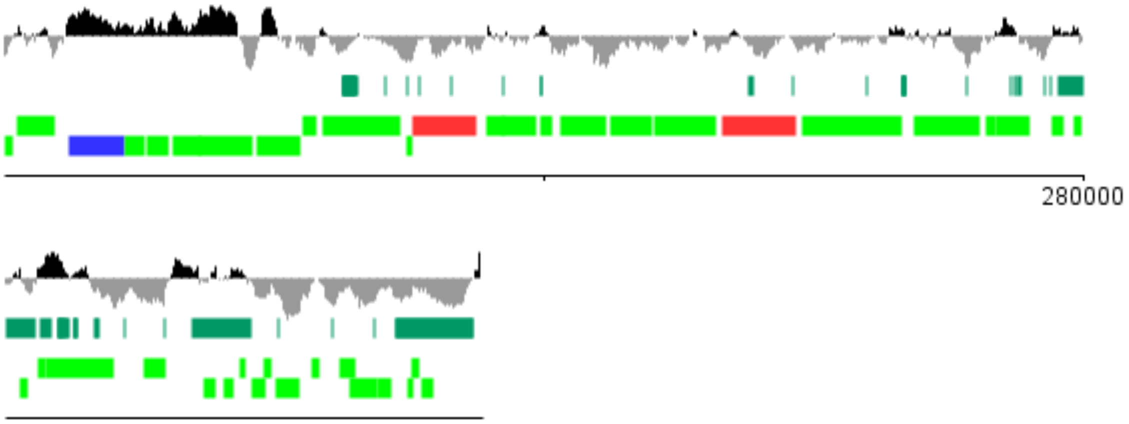
280000
