# Supplementary material for: The End of a 60-year Riddle: Identification and Genomic Characterization of an Iridovirus, the Causative Agent of White Fat Cell Disease in Zooplankton
Source: G3 (Bethesda). 2018 Feb 27;8(4):1259–72. doi: 10.1534/g3.117.300429 (PMC5873915; doi:10.1534/g3.117.300429)
Supplement: Supplementary file 7 [file 1259TableS5.docx]

**Supplemental Table S5.** Additional proposed core genes by Eaton *et al.* (2007). Clustering analyses of orthologous groups of proteins based on RBH using four different cutoff settings.

| **Putative function** | **IIV-6 proteins** | **DIV-1 proteins** | **Orthologous cluster^a^ (alg. conn.^e^ in %)** | **Orthologous cluster^b^ (alg. conn. in %)** | **Orthologous cluster^c^ (alg. conn. in %)** | **Orthologous cluster^d^ (alg. conn. in %)** |
| --- | --- | --- | --- | --- | --- | --- |
| **serine/threonine protein kinase** | 380R (NP_149843.1) | no orthologue | - | - | - | 12.4 |
| **lipopolysaccharide-modifying enzyme/ tyrosine kinase** | 179R (NP_149642.1) | no orthologue | - | - | 10.9 | 10.9 |
| **uncharacterized protein** | 067R (NP_149530.1) | no orthologue | - | - | - | 90.6 |
| **proliferating cell nuclear antigen** | 436R (NP_149899.1) | no orthologue | - | - | 59.3 (IIVs)/ 92.6 (VIVs) | 59.3 (IIVs)/ 92.6 (VIVs) |

^a^clustering analysis 1 (coverage 30 %, identity 30 %, minimal connectivity 10 %), ^b^clustering analysis 2 (coverage 30 %, identity 30 %, minimal connectivity 5 %), ^c^clustering analysis 3 (coverage 20 %, identity 20 %, minimal connectivity 10 %), ^d^clustering analysis 4 (coverage 20 %, identity 20 %, minimal connectivity 5 %), ^e^algebraic connectivity

**Supplemental reference**

Eaton HE, Metcalf J, Penny E, Tcherepanov V, Upton C, Brunetti CR. 2007. Comparative genomic analysis of the family *Iridoviridae*: re-annotating and defining the core set of iridovirus genes. *Virol J* **4**: 11.
