# Supplementary material for: The End of a 60-year Riddle: Identification and Genomic Characterization of an Iridovirus, the Causative Agent of White Fat Cell Disease in Zooplankton
Source: G3 (Bethesda). 2018 Feb 27;8(4):1259–72. doi: 10.1534/g3.117.300429 (PMC5873915; doi:10.1534/g3.117.300429)
Supplement: Supplementary file 9 [file 1259TableS7.docx]

**Supplemental Table S7.** Orthologous proteins conserved in 90% of all IIVs. Clustering analyses of orthologous groups of proteins based on RBH using four different cutoff settings.

| **Putative function** | **IIV-6 proteins** | **DIV-1 proteins** | **Orthologous cluster^a^ (alg. conn.^e^ in %)** | **Orthologous cluster^b^ (alg. conn. in %)** | **Orthologous cluster^c^ (alg. conn. in %)** | **Orthologous cluster^d^ (alg. conn. in %)** |
| --- | --- | --- | --- | --- | --- | --- |
| **Orthologous clusters conserved in all IIVs, but not found in DIV-1** | | | | | | |
| **exonuclease** | 012L (NP_149475.1) | no orthologue | **100** | **100** | **100** | **100** |
| **DNA topoisomerase 2** | 045L (NP_149508.1) | no orthologue | **100** | **100** | **100** | **100** |
| **uncharacterized protein** | 050L (NP_149513.1) | no orthologue | - | - | 52.4 | 52.4 |
| **uncharacterized protein** | 056R (NP_149519.1) | no orthologue | - | - | - | 8.8 |
| **uncharacterized protein** | 111R (NP_149574.1) | no orthologue | **19.2** | **19.2** | **54.5** | **54.5** |
| **uncharacterized protein** | 115R (NP_149578.1) | no orthologue | **42.1** | **42.1** | **100** | **100** |
| **uncharacterized protein** | 122R (NP_149585.1) | no orthologue | - | - | 49.9 | 49.9 |
| **uncharacterized protein** | 141R (NP_149604.1) | no orthologue | - | - | 16.5 | 16.5 |
| **uncharacterized protein** | 145L (NP_149608.1) | no orthologue | - | - | 10.5 | 10.5 |
| **uncharacterized protein** | 196R (NP_149659.1) | no orthologue | **100** | **100** | **100** | **100** |
| **tyrosine phosphatase** | 197R (NP_149660.1) | no orthologue | **63.6** | **63.6** | **100** | **100** |
| **uncharacterized protein** | 218R (NP_149681.1) | no orthologue | - | - | 13.5 | 13.5 |
| **uncharacterized protein** | 219L (NP_149682.1) | no orthologue | - | - | - | 9.74 |
| **ubiquitin thioesterase** | 232R (NP_149695.1) | no orthologue | - | - | 13.6 | 13.6 |
| **uncharacterized protein** | 234R (NP_149697.1) | no orthologue | **15.2** | **15.2** | **63.6** | **63.6** |
| **phosphoesterase** | 244L (NP_149707.1) | no orthologue | **100** | **100** | **100** | **100** |
| **uncharacterized protein** | 250L (NP_149713.1) | no orthologue | **15.3** | **13.5** | **13.5** | **13.5** |
| **zinc finger protein** | 302L (NP_149765.1) | no orthologue | - | - | 38.7 | 38.7 |
| **SWIB domain-containing protein** | 306R (NP_149769.1) | no orthologue | **100** | **100** | **100** | **100** |
| **uncharacterized RING finger protein** | 332L (NP_149795.1) | no orthologue | - | - | - | 14.4 |
| **uncharacterized protein** | 335L (NP_149798.1) | no orthologue | - | - | 100 | 100 |
| **uncharacterized protein** | 358L (NP_149821.1) | no orthologue | **15.3** | **15.3** | **72.7** | **72.7** |
| **uncharacterized protein** | 359L (NP_149822.1) | no orthologue | - | - | 19.8 | 19.8 |
| **uncharacterized protein** | 391R (NP_149854.1) | no orthologue | **100** | **100** | **100** | **100** |
| **proliferating cell nuclear antigen** | 436L (NP_149899.1) | no orthologue | - | - | 59.3 | 59.3 |
| **uncharacterized protein** | 467R (NP_149930.1) | no orthologue | **10.5** | **10.5** | **100** | **100** |
| **uncharacterized protein** | 082L (NP_149545.1) | no orthologue | - | 8.62 | 100 | 100 |
| **uncharacterized protein** | 192R (NP_149655.1) | no orthologue | **40** | **40** | **40** | **40** |
| **RING finger protein** | 413R (NP_149876.1) | no orthologue | - | 6.58 | - | - |
| **Orthologous clusters conserved in 90% of all IIVs, including DIV-1.** | | | | | | |
| **DNA helicase Pif1-like** | 030L (NP_149493.1) | DIV1_077R | - | - | 100 | 100 |
| **uncharacterized protein** | 198R (NP_149661.1) | DIV1_255L | - | - | (100, cluster with all IIVs) | (100, cluster with all IIVs) |
|  |  | no orthologue | 17.3 | 17.3 | - | - |
| **RNA polymerase subunit RPB5** | 454R (NP_149917.1) | DIV1_147L | - | - | 63.6 | (7.51, cluster with all IVs) |
|  |  | no orthologue | - | 6.39 | - | - |
| **uncharacterized protein** | 155L (NP_149618.1) | DIV1_157R | - | - | 72.7 | (6.71, cluster with all IIVs) |
|  | 155L (NP_149618.1), 149L (NP_149612.1) | no orthologue | - | 6.77 | - | - |
| **ribonuclease H-like** | 170L (NP_149633.1) | DIV1_195R | - | - | 63.6 | - |
|  |  | no orthologue | 18.2 | 18.2 | - | - |
| **DNA ligase** | 205R (NP_149668.1) | DIV1_273L | - | - | (100, cluster with all IIVs) | (100, cluster with all IIVs) |
|  |  | no orthologue | 100 | 100 | - | - |
| **uncharacterized protein** | 268L (NP_149731.1) | DIV1_256R | - | - | (41.7, cluster with all IIVs) | (41.7, cluster with all IIVs) |
|  |  | no orthologue | 15.3 | 15.3 | - | - |
| **uncharacterized protein** | 309L (NP_149772.1) | DIV1_165R | - | - | (41.7, cluster with all IIVs) | (41.7, cluster with all IIVs) |
|  |  | no orthologue | 16.9 | 16.9 | - | - |
| **uncharacterized protein** | 325L (NP_149788.1), 203L (NP_149666.1) | DIV1_272L | - | - | - | (9.13, cluster with all IIVs) |
|  | 325L (NP_149788.1) | no orthologue | 17.3 | 17.3 | - | - |
| **uncharacterized protein** | 357R (NP_149820.1) | DIV1_187L | - | - | - | (8.33, cluster with all IIVs) |
|  |  | no orthologue | 10.5 | 10.5 | - | - |
| **uncharacterized protein** | 378R (NP_149841.1) | DIV1_025L, DIV1_173R | - | - | - | (7.57, cluster with all IIVs) |
|  |  | no orthologue | 100 | 100 | - | - |
| **helicase** | 184R (NP_149647.1) | DIV1_078R | - | - | (84.6 cluster with all IVs) | (84.6 cluster with all IVs) |
|  |  | no orthologue | 100 | (6.09 cluster with all IVs) | - | - |
| **thymidine kinase protein** | 143R (NP_149606.1) | DIV1_308L | **-** | **-** | (85.1, cluster with all IVs) | (85.1, cluster with all IVs) |
|  |  | no orthologue | 100 | 100 | - | - |
| **uncharacterized protein** | 287R (NP_149750.1) | DIV1_328R | - | - | (63, cluster with almost all IVs) | (63, cluster with all IVs) |
|  |  | no orthologue | 54.5 | 54.5 | - | - |

^a^clustering analysis 1 (coverage 30 %, identity 30 %, minimal connectivity 10 %), ^b^clustering analysis 2 (coverage 30 %, identity 30 %, minimal connectivity 5 %), ^c^clustering analysis 3 (coverage 20 %, identity 20 %, minimal connectivity 10 %), ^d^clustering analysis 4 (coverage 20 %, identity 20 %, minimal connectivity 5 %), ^e^algebraic connectivity.
